# Neural Network Models for Prediction of Biological Activity using Molecular Dynamics Data: A Case of Photoswitchable Peptides

**DOI:** 10.1002/minf.70001

**Published:** 2025-07-14

**Authors:** Anton Cherednichenko, Sergii Afonin, Oleg Babii, Taras Voitsitskyi, Roman Stratiichuk, Ihor Koleiev, Volodymyr Vozniak, Nazar Shevchuk, Zakhar Ostrovsky, Semen Yesylevskyy, Alan Nafiiev, Serhii Starosyla, Anne S. Ulrich, Aigars Jirgensons, Igor V. Komarov

**Affiliations:** ^1^ Taras Shevchenko National University of Kyiv Kyiv Ukraine; ^2^ Enamine Ltd Kyiv Ukraine; ^3^ Karlsruhe Institute of Technology (KIT) Karlsruhe Germany; ^4^ Receptor.AI Inc. London UK; ^5^ Institute of Physics of The National Academy of Sciences of Ukraine Kyiv Ukraine; ^6^ Institute of Organic Chemistry and Biochemistry Czech Academy of Sciences Czech Republic; ^7^ Palacký University Olomouc Olomouc Czech Republic; ^8^ Latvian Institute of Organic Synthesis Riga Latvia

**Keywords:** biological activity, diarylethenes, molecular dynamics, neuronal network models, photoswitchable peptides

## Abstract

Prediction of biological activities of chemical compounds by the machine learning techniques in general and the neural networks (NNs) in particular, is usually based on the analysis of their binding to the target of interest. If such affinity data is not available, the ligand‐based approaches can be used where the NN models are trained to assess similarity of compounds to those with known biological activity. Obviously, this approach only works well if the similarity between the training set and the evaluated molecules is sufficiently high. In the case of large and conformationally flexible organic compounds, the activity becomes dependent not only on chemical identity but also on the dynamics of molecular motions, which imposes significant challenges to existing approaches based on static structural 2D and 3D molecular descriptors. A prominent example of compounds, which are especially challenging for existing NN activity prediction techniques, are photoswitchable macrocyclic peptides containing a diarylethene “photoswitch” (DAE). These molecules exist in two isomeric forms with remarkably different biological activities, which are interconvertible by light of different wavelengths. Activity prediction models have to distinguish in this case not only between the different peptides but also between the photoisomers of the same peptide. In this work, we demonstrate that the features extracted from classical molecular dynamics (MD) trajectories are superior to conventional 2D or 3D descriptor‐based features when used in activity prediction NN models of DAE‐containing photoswitchable peptides. Using MD‐derived features, we successfully created two NN models that predict activities of photoswitchable peptidomimetics, analogs of the natural peptidic antibiotic gramicidin S. The first model precisely predicts the cytotoxic activity of similar peptide analogs. The second model reliably predicts the differences in the biological activities of DAE photoisomers of the same peptide, even if the type of its activity differs from one in the training dataset. Our results demonstrate that accounting for MD‐derived dynamic features allows generalizing the ligand‐based activity prediction NN models to the cases of large and conformationally flexible molecules, which were previously considered intractable by this class of models.

## Graphical abstract

Two neural network models are reported that can reliably predict biological activities of complex molecules like cyclic peptides in target‐independent manner. Both models use the features extracted from molecular dynamics simulation data and contain information about the 3D structure and dynamics of the molecules.

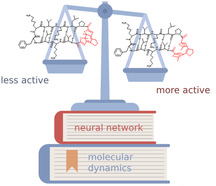

## 1 Introduction

The last decade has witnessed unprecedented growth of machine learning (ML) and artificial intelligence (AI) systems for drug discovery [1, 2, 3, 4–5]. In particular, neural network (NN) models become increasingly popular for predicting the properties of drugs and therapeutically relevant compounds [6, 7]. Most of these models are trained using the data describing the interaction of compounds with their biological targets.

The biological activity of chemical compounds is tied to specific biological targets, but it can also be predicted in a target‐independent manner using the ligand‐based techniques, such as the NN models. These models, based on quantitative structure–activity relationships (QSAR), rely on the principle that structurally similar molecules are likely to exhibit similar biological effects [8]. This approach simplifies the task of comparing novel compounds with those in existing libraries that have well‐documented biological properties. The effectiveness of these predictions largely depends on the molecular descriptors used to measure similarity [9]. An established approach in cheminformatics is analysis of neighborhood behavior (NB)—a relationship between the structural dissimilarity metric of the calculated molecular descriptor space and the activity dissimilarity metric [10, 11].

Most descriptors used to train and optimize NN models for similarity searches are derived from atomic connectivity, making them 2D by nature [12]. Popular examples include substructure‐based fingerprints and topological and circular fingerprints as well as pharmacophore fingerprints [13]. These 2D descriptors have proven highly effective for predicting biological activity, especially for small molecules, and are a cornerstone of modern AI‐driven drug discovery [14].

2D fingerprints often fall short when dealing with large, structurally complex compounds. This challenge has led researchers to focus on 3D descriptors, which are particularly effective for capturing the intricate features of complex biomolecules [15]. For such cases, a promising approach is to further enhance NN models by incorporating molecular features that reflect not only 3D structural information but also molecular dynamics (MD) and flexibility. The energy associated with the flexibility and conformational dynamics of ligand molecules plays a crucial role in their binding interactions with targets, significantly influencing their biological activity [16, 17–18].

In this study, we present our findings on developing NN models for predicting the biological activity of macrocyclic peptides using both structural and dynamic data derived from MD simulation trajectories. We hypothesized that MD trajectories of unbound compounds provide sufficient structural and dynamic information for NN models to accurately predict the activity of relatively large and flexible molecules, such as peptides. It is worth noting that MD simulations are widely used to refine predictions from sequence‐based NN models for peptides [19]; however, the use of models trained exclusively on MD simulation data remains relatively uncommon [20] (see, however [21, 22]).

**FIGURE 1:**
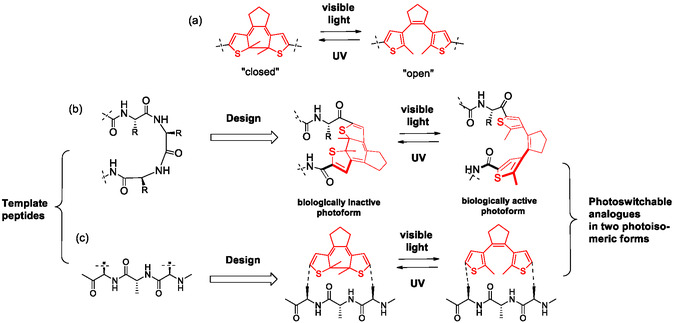
(a) A diarylethene photoswitch, its photoisomerization and isomer nomenclature; (b) modification of a peptide backbone by a diarylethene fragment; (c) side‐chain “stapling” of the peptide by a diarylethene fragment.

Our motivation in developing QSAR models for bioactive peptides is driven by our prior research on the design and synthesis of compounds whose biological activity can be modulated by light. The design begins with a natural or synthetic biologically active peptide (template), which is modified by incorporating a photoisomerizable fragment or “molecular photoswitch.” For this purpose, we used a diarylethene (DAE) photoswitch [23], capable of interconverting between two photoisomeric states—“closed” and “open”—when exposed to light of different wavelengths, as depicted in Figure 1a.

In our approach, one or more amino acid residues in the template peptide are replaced with the DAE fragment. This design can ensure that the resulting analog retains the biological activity of the original peptide in one photoisomeric form, while photoconversion to the other form leads to a loss of activity (Figure 1b). Alternatively, the photoisomerizable fragment can be integrated into a side chain of the template peptide or used to cross‐link (“staple”) two side chain residues, as illustrated in Figure 1c. This versatility in design allows for fine‐tuning the photoresponsive behavior of the peptide analogs.

Similar approaches have been developed for other types of known photoisomerizable fragments, for example, “azologization” for azobenzene derivatives [24, 25], or “stapling” for spiropyran photoswitches [26]. In general, there are many possible ways to modify the template with photoswitches, and it is not obvious a priori which modification will lead to the best‐performing photoswitchable analog. As the synthesis of photoswitchable analogs is usually resource‐consuming, theoretical models that can predict the effect of the modification are highly desirable.

## 2 Methods

### 2.1 General Strategy for Constructing the NN Models

**FIGURE 2:**
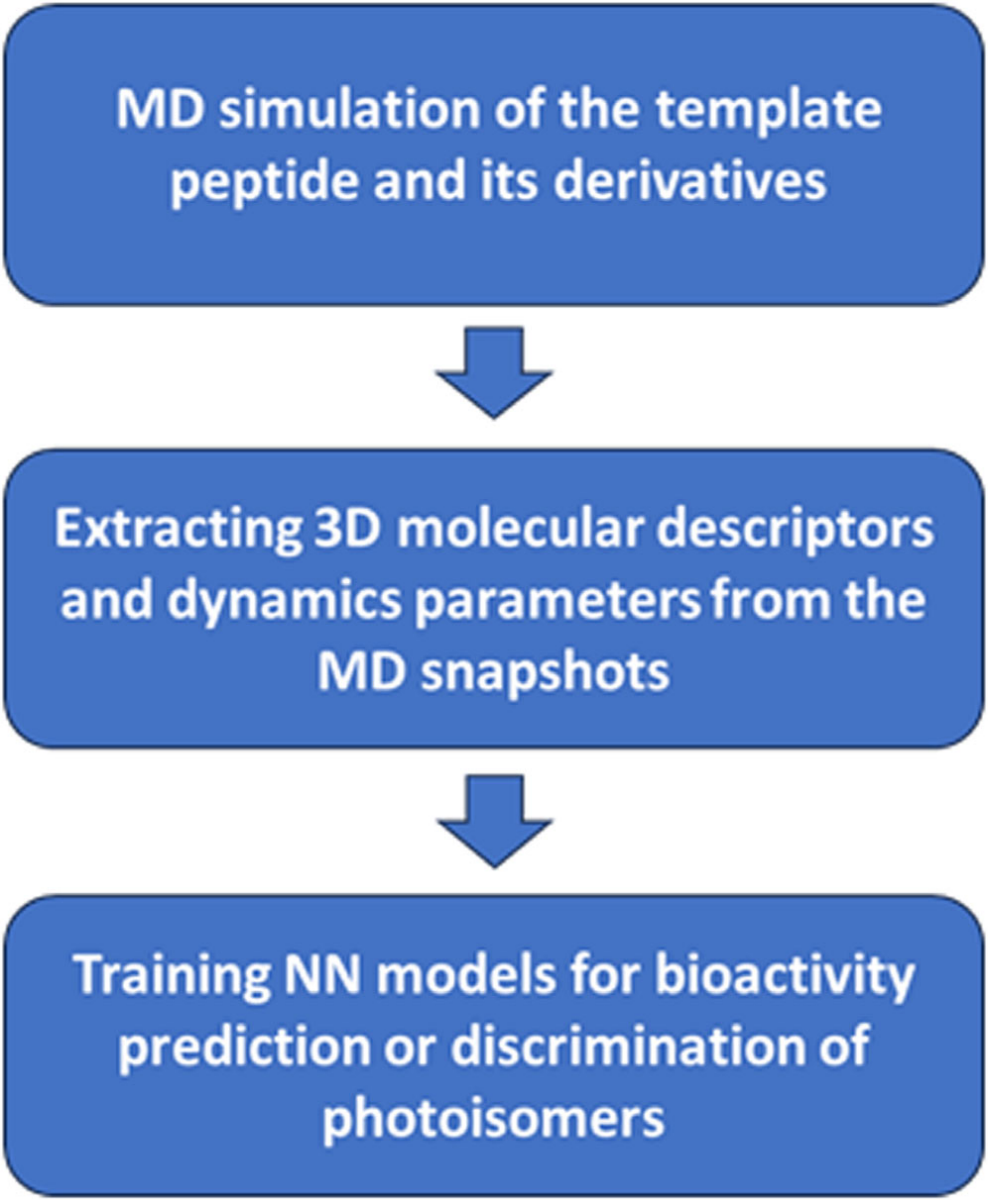
Strategy for NN construction used in this work.

Encouraged by the work of Prakash et al. [27] on building a NN model for the rational design of antimicrobial peptides, we adopted a similar approach. Prakash and coworkers utilized simple parameters derived from short MD simulations of synthetic cationic peptides known as CAMEL‐s [28] to develop their model and demonstrate its utility. Among the parameters they used, the average volume of the peptide molecules and their solvent accessible surface area were shown to significantly influence antimicrobial activity, while an average number of hydrogen bonds, ellipticity, and moment of inertia were found less critical. Building on these insights, we incorporated 3D descriptors calculated from the MD snapshots into our models. We believe that incorporating both 3D structure‐ and dynamics‐based parameters allows for the construction of mechanism‐independent models of general applicability. Our strategy for constructing the NN models is outlined in Figure 2.

### 2.2 Compound Set

**FIGURE 3:**
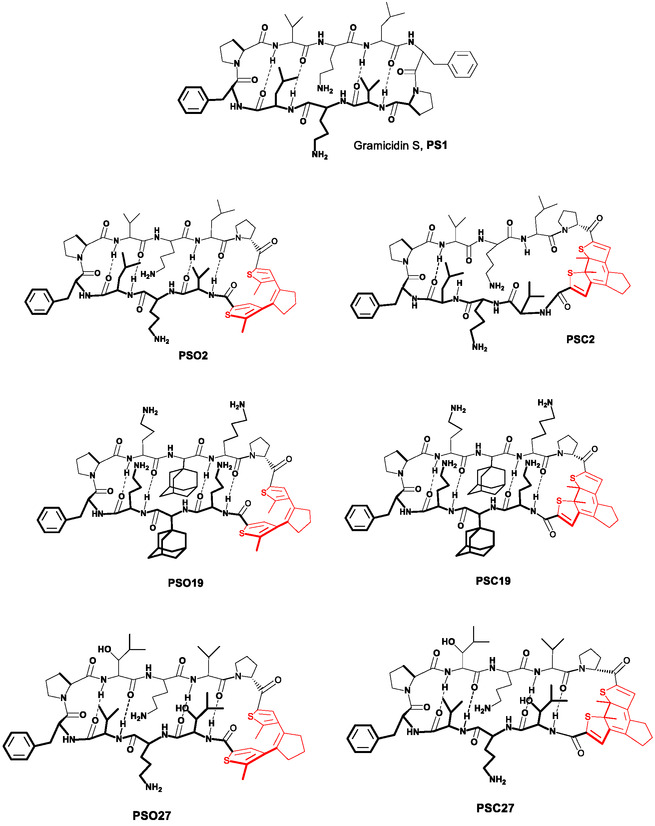
The peptide‐prototype, natural cytotoxic antibiotic gramicidin S and some of its diarylethene‐containing photoswitchable analogs used to build the predictive NN models in this work (photoisomerizable diarylethene fragment is shown in red). For the complete set of compounds, see Supporting Information.

The peptide set and experimental data used to build our models and perform analyses were synthesized and tested for cytotoxic activity earlier by our group during a search for the most efficient photoswitching diarylethene‐containing analogs of the natural cytotoxic peptide gramicidin S [29]. Previous studies have demonstrated that the activity of the DAE‐derived gramicidin S analogs and similar cyclic peptides depends not only on their structure but also on the dynamics of the molecules [30]. Therefore, the use of MD simulation data for predictive models is particularly justified for this class of compounds. For this study, we selected 26 photoswitchable gramicidin S analogs described in [29], featuring the photoswitch in both open and closed photoforms. The cytotoxic activity of all these peptides was also reported in the same study, with minimal growth inhibitory concentrations (MIC) values against Escherichia coli and Bacillus subtilis, 50% toxicity concentrations (IC_50_) against HeLa cell line, and 50% hemolysis (HC_50_) concentrations for human erythrocytes determined. We used the IC_50_ values for training and validating our models. Figure 3 displays the structural formulas of gramicidin S and some of its photoswitchable analogs; for the complete set of formulas (gramicidin S (PSO1), PSO2−26 (“O” means “open photoform”, the number will be used as compound ID in the following text),^1^ PSC2−26 (“C” means ”closed photoform”, compound ID in the following text)^2^ and corresponding IC_50_ values, see the Supporting Information.

### 2.3 NN Models

For each compound, including gramicidin S and all its analogs in both photoisomeric forms, we conducted MD simulations as described in the Supporting Information. Although Prakash et al. reported enhanced performance of their model when simulation data in octanol were used [27], we performed all our calculations in water. This decision was made because the exact mechanisms of action for all compounds were not known, and we aimed for a generalized approach. Our objective was to differentiate between the MD trajectories of gramicidin S and those of the derivatives containing either a closed or open photoswitch. To this end, two models were constructed, each addressing different aspects of this issue.

The first model, termed the “Cytotoxicity Prediction Model,” was designed to predict the cytotoxicity of peptides (specifically, the values of IC_50_ in µM) by analyzing each frame of the MD trajectory. The primary concept of this model is to predict the “activities” for the conformations at each frame (regression value IC_50_) and then compute the mean value of these proto‐IC_50_ values over all MD frames for each compound to get the final IC_50_ assessment. We focused in this model on 10 000 frames. We took an equilibrated part of a trajectory and selected 10 000 frames by clustering on 10 000 clusters and picking one representative conformation from each cluster. The agglomeration algorithm from the Scikit‐Learn Python library was used [31].

By using this approach, we can compare different pairs of trajectories for their similarity—for instance, the open and closed forms, or any pair of the peptides, including the original template peptide. By conducting three sets of comparisons—open‐closed, open‐template, and closed‐template—we can assess the effectiveness of inserting the diarylethene fragment into the peptide and its photoswitching capabilities. The ideal scenario occurs when the open and closed forms exhibit distinct activity values, whereby the form generated by visible light (open) is close in activity to the template. In such cases, the activity can be “switched on” in biological systems, even in living tissues of higher organisms, because visible light penetrates tissues better than UV light [32].

From a practical standpoint, it is crucial for a particular photoswitching derivative to determine whether the isomerization of the photoswitch would affect the biological activity. Only those derivatives that demonstrate efficient photocontrol of biological activity are promising for use as drugs or research tools. Consequently, we developed the second model, called the “Isoform Similarity Model.” Unlike the Cytotoxicity Prediction Model, the main objective of Isoform Similarity Model is to generalize the process of identifying differences between the open and closed forms across various DAE‐modified peptides, including those with biological activities not limited to anti‐HeLa cytotoxic action. To achieve this, a binary classification model was developed to estimate similarity of a peptide open‐closed isomeric pair, in essence, compute their NB‐compliance with respect to their in vitro activity values. This model enables assessment of similarity in this sense between the closed and open forms, expressed as a binary value: Class 1 if there is a significant difference in IC_50_ values and Class 0 if there is no such difference. Statistically, it was determined that the threshold for the ratio between the IC_50_ activities of the closed and open forms is five, for them to demonstrate good NB. Pairs (open‐closed forms) with ratios less than five were considered to exhibit no significant difference in IC_50_ activities. Since we were analyzing pairs of open‐closed forms in this model, each training sample was considered a concatenated feature vector for each open and closed MD trajectory frame.

We have 260 000 samples (26 pairs of closed‐open forms, each with 10 000 frames) represented by a vector of 8 192 features (two E3FP vectors, each with 4 096 features).

As in the Cytotoxicity Prediction Model, when splitting the data into cross‐validation and test sets, we ensured that all frames of one pair of trajectories went into the same data set. Additionally, we needed to maintain class balance. Table S5, Supporting Information, provides information on the molecules which were included in the training and validation (test) sets. Table S6 (Supporting Information) contains information about each split, including the number of samples and the percentage of the total data it represents.

### 2.4 Experimental Data

Each MD trajectory for all 26 diarylethene‐containing compound pairs was represented by 10 000 frames, which were interpreted as a single input sample for the model. Therefore, our dataset comprised 520 000 samples, with a strict association with the open or closed form to a particular peptide when forming train‐test splits for both models. Each specific dataset, whether a validation or a test set, included both closed and open forms for each gramicidin S analogue.

## 3 Results and Discussion

### 3.1 Cytotoxicity Prediction Model

For the regression value we aimed to predict by this model, we used the natural logarithm of the HeLa cytotoxic half‐activity values expressed in micromoles/L (ln(IC_50_)), obtained experimentally [29]. Details for the labels and compound set split used for the Cytotoxicity Prediction Model are provided in Tables S1–S3 (Supporting Information).

We performed several experiments to find the best possible features and model. We tested and compared in these experiments two main model families: the default Random Forest regressor from Scikit‐Learn with a max_depth parameter equal to 10 to avoid overfitting and standard Multi‐Layer Perceptron (MLP) with the following architecture: one hidden layer with two times more hidden units than used for the input, batch normalization layers and dropout layer with 0.1 dropout probability, ReLU activation function, Adam optimizer with 0.001 learning rate and 0.0005 weight decay and 256 batch size. We also compared the final predictions obtained with the use of 2D descriptors with the results obtained with 3D descriptors. For this comparison, we choose the most popular 2D fingerprints from RDKit (Morgan with a radius 2 and 1024 bits; Avalon, Atom and Topological with 1024 bits; RDK and Layered with a max path 3 and 1024 bits; MACCS keys). As for the 3D descriptors, we tested Mordred 3D descriptors and E3FP features with 4096 bits. Also, we tried to fetch MD features for every trajectory frame including the RMSD (root mean square deviation), inertia tensor, radius of gyration of the peptides calculated by the mdtraj Python library [33], and polar surface area (PSA) calculated by RDKit [34]. Table 1 summarizes all the features used for the Cytotoxicity Prediction Model in different experiments. As the metric for evaluation and experiment comparison, we chose R‐squared. Some experiments included an extra step, the feature selection. We selected the most valuable features for the model using a variance threshold from Scikit‐Learn with a threshold value of 0.075. For the 2D fingerprints experiment (2D Fingerprints + MLP + Feature Selection), the following features were selected: 44 Morgan, 413 Avalon, 333 Atom, 44 RDK, 56 Topological, 28 Layered fingerprints, and 16 MACCS keys. For the best experiment (Mordred Features + MD Features + E3FP Fingerprints + MLP + Feature Selection), the following features were selected: 7 MD features (6 for inertia tensor and 1 for PSA), 38 Mordred 3D features, and 483 E3FP fingerprints. Table 2 shows the results of all the experiments for both cross‐validation (CV) and test set.

**TABLE 1:** Overview of the features used for the Cytotoxicity Prediction Model.

Feature	**Number of features**
Morgan fingerprints	1024
Avalon fingerprints	1024
Atom fingerprints	1024
Topological fingerprints	1024
RDK fingerprints	1024
Layered fingerprints	1024
MACCS keys	167
Mordred 3D descriptors	51
MD features	9

**TABLE 2:** Overview of the experiments performed in search for the best Cytotoxicity Prediction Model.

Experiment	Number of features	R‐Squared (CV)	R‐Squared (Test)
Morgan fingerprints + Random Forest	1024	0.394	0.346
E3FP fingerprints + Random Forest	4096	0.349	0.319
Mordred features + MD features + Random Forest	60	0.424	0.402
Morgan fingerprints + MLP	1024	0.528	0.112
2D Fingerprints + MLP	6311	0.621	0.119
2D Fingerprints + MLP + Feature Selection	934	0.653	0.132
E3FP fingerprints + MLP	4096	0.467	0.343
Mordred features + MD Features + MLP	60	0.669	0.574
Mordred Features + MD Features + E3FP Fingerprints + MLP	4156	0.552	0.855
2D Fingerprints + Mordred Features + MD Features + E3FP Fingerprints + MLP	10 467	0.655	0.146
**Mordred Features + MD Features + E3FP Fingerprints + MLP + Feature Selection**	**528**	**0.672**	**0.753**
2D Fingerprints + Mordred Features + MD Features + E3FP Fingerprints + MLP + Feature Selection	1462	0.664	0.154

The following conclusions can be done after analyzing the results of the experiments:

• 3D descriptors are much more effective than 2D descriptors for our purpose;
• MLP is a better choice rather than classic ML models;
• The best experiment uses a combination of 3D descriptors plus feature selection. Therefore, for tuning, we used MLPs with the 528 input features described above.

Hyperparameters for the tuning included:

• Number of layers: from 1 to 4;
• Optional batch normalization: one of [True, False] (the same value for all layers);
• Dropout rate: from 0.1 to 0.5 (the same value for all layers);
• Number of hidden features: one of [264, 528, 1056, 1584] (on each layer).

The following parameters were also considered as hyperparameters (for an optimizer and batch size):

• Learning rate: one of [1e‐4, 1e‐3, 1e‐2];
• Weight decay: one of [5*1e‐5, 5*1e‐4, 5*1e‐3];
• Batch size: one of [16, 32, 64, 128, 256].

The parameters that remained fixed included the ReLU activation function on each layer and the Adam [35] optimizer.

The tuning was done using the Python library Optuna [36], using a Bayesian approach for hyperparameter optimization. The R‐squared was selected as the evaluation metric for optimization. The tuning process aimed to maximize this metric when choosing the best architecture. A total of 100 trials were conducted; early stopping was applied, set to 20 trials (if Optuna did not find an improvement in the evaluation metric within 20 trials, the tuning would conclude without reaching the 100 initially designated trials). The tuning process resulted in 83 trials, with the best score found in the 63rd trial.

The optimal architecture consisted of 1 layer with 120 hidden features, batch normalization, a dropout rate of 0.194, a 0.001 learning rate, a 0.0005 weight decay, and a 256 batch size. Table 3 provides the architecture of the final optimised Cytotoxicity Prediction Model.

**TABLE 3:** The best MLP architecture for the Cytotoxicity Prediction Model.

Layer	Output shape	Parameters
BatchNorm1D	[1, 528]	1 056
Linear	[1, 1056]	558 624
BatchNorm1D	[1, 1056]	2 112
ReLU	[1, 1056]	—
Dropout	[1, 1056]	—
Linear	[1, 1]	1 057

As a result of this approach, the model can reliably predict the IC_50_ against HeLa cell line for gramicidin S and its DAE‐modified analogs in any form (open, closed). Consequently, by analyzing different pairs of peptides (open‐closed, closed‐original, and open‐original), it is possible to predict the difference between the closed and open forms and draw conclusions regarding the impact of the photoswitching on peptide activity.

Table 4 provides metrics for per‐frame results (“per‐frame” means that each trajectory frame was considered as an individual input sample for the model, the activity for this metric was predicted for each frame as an intermediate value).

**TABLE 4:** Metrics for the Cytotoxicity Prediction Model (per frame).

Metric	CV	Test
MSE	0.498	0.257
MAE	0.521	0.420
MAPE	0.212	0.123
R‐squared	0.693	0.781

**FIGURE 4:**
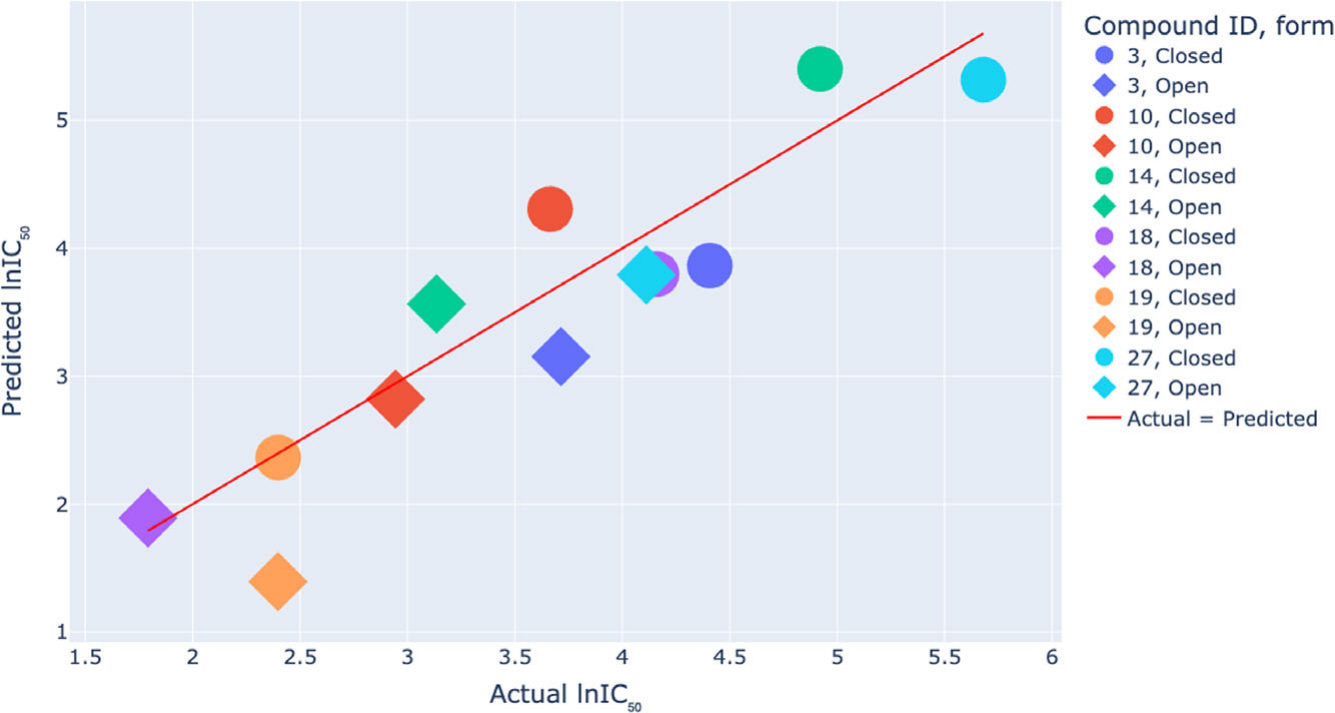
Relationship between actual and predicted IC_50_ values (µM against HeLa cell line) of trajectories for the test set for the Cytotoxicity Prediction Model.

We were interested in the overall activity in the outcome, over the whole trajectory, which we determined as the averaged result across all MD frames for each compound. For this purpose, we used the aggregation scheme described previously as multi‐instance ML approach [37]. The correlation of the averaged values with the experimental activity values for the test set is depicted in Figure 4. This figure highlights the closed and open forms of the same derivative trajectory with matching colours (closed forms are circles and open forms are diamonds). As can be seen, this version of the model can accurately predict the difference in anti‐HeLa IC_50_ between closed and open forms of the backbone‐modified diarylethene‐derived gramicidin S analogues. Even if the model fails to precisely predict biological activity (as in the case of compound PSO19), it still correctly captures the actual difference between the closed and open photoisomers.

**FIGURE 5:**
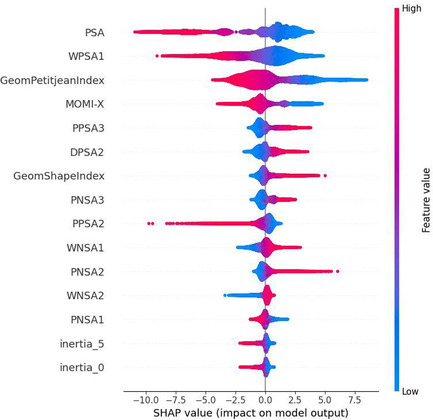
Beeswarm plot for the distribution of SHAP values for top 15 features for the Cytotoxicity Prediction Model.

**FIGURE 6:**
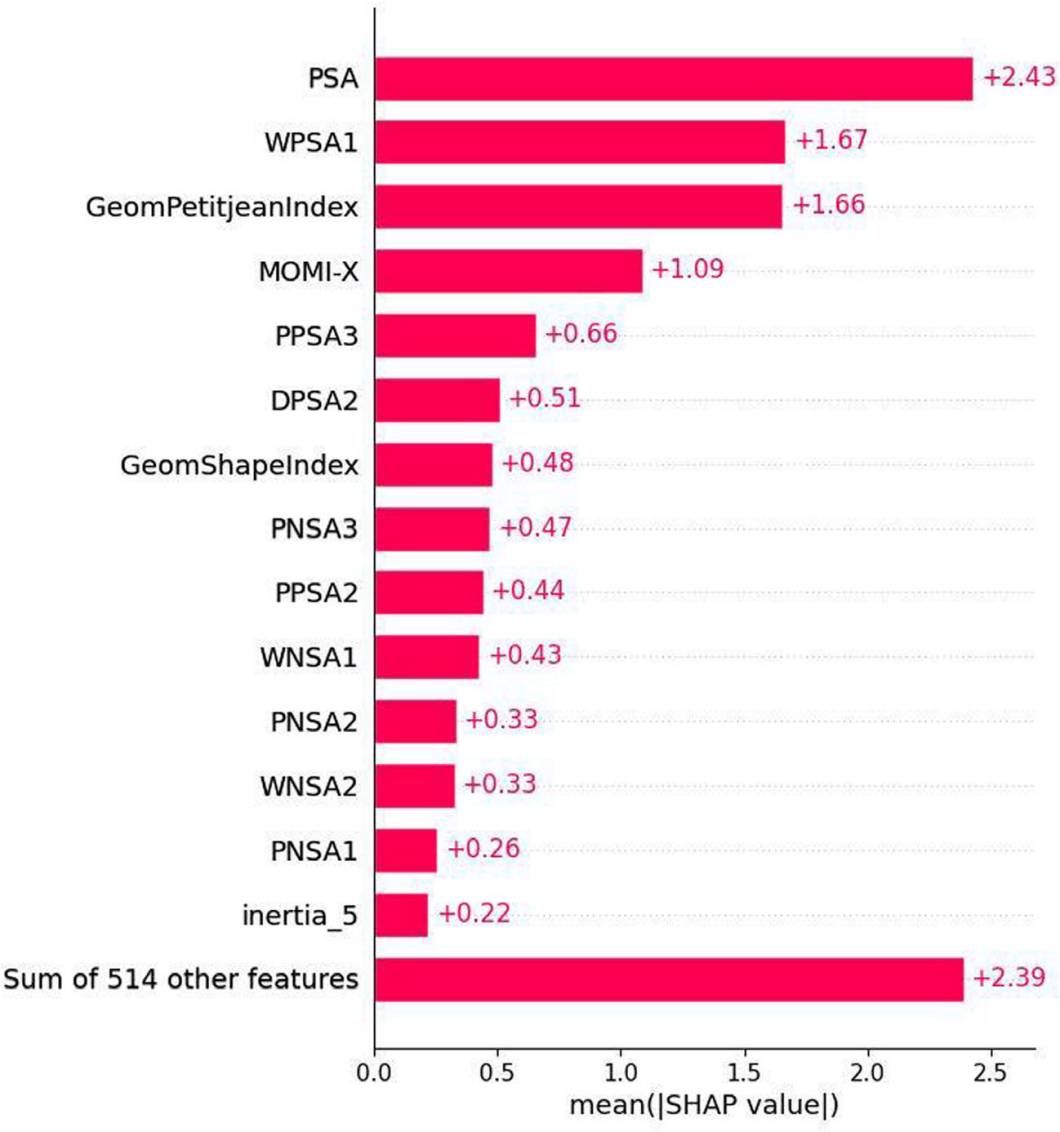
Bar plot with mean absolute SHAP value of each feature over all the instances in the test set for the Cytotoxicity Prediction Model.

We estimated that the best descriptors which make the largest impact for the predictive value of this model are 3D Mordred descriptors, MD features, and E3FP fingerprints. For this estimation, we used SHAP values, calculating them by basic explainer from the SHAP Python library for samples from the test set. This analysis is similar to the NB approach, which is based on plots in coordinates activity difference‐descriptor dissimilarity values in a chemical space [10].^3^ Figure 5 shows a beeswarm plot that summarizes the entire distribution of SHAP values for top 15 features. Features are sorted by the sum of the SHAP value magnitudes across all samples. Figure 6 shows a bar plot with mean absolute SHAP value for each feature over all the instances in the test set. It is interesting to note that Mordred 3D descriptors and some molecular dynamic features (values of inertia tensor) have the most significant impact on the final predictions while individual E3FP fingerprints do not have such an impact. E3FP are binary descriptors while other 3D descriptors are continuous. 3D Modred/MD descriptors having a greater number of distinct values are more sensitive to perturbations than E3FP. Since SHAP approach is based on descriptor perturbations, 3D descriptors will receive greater contributions. But combinations of different bits give useful information for the model as adding them boosts the model's performance. This can also be confirmed with some numerical values for E3FP fingerprints: the sum of mean absolute SHAP values of all E3FP fingerprints is equal to 0.77, which is comparable with other descriptors in Figure 5, but the individual E3FP fingerprint has the average impact (mean absolute SHAP value) of 0.0016, which is quite small.

### 3.2 Isoform Similarity Model

First, it is important to note that in this approach, we consider pairs of closed and open forms as the primary entries, rather than analyzing each trajectory individually as was in the Cytotoxicity Prediction Model. Second, to obtain labels for Isoform Similarity Model, it was necessary to determine a potential cutoff for the activity values ratio between closed and open anti HeLa IC_50_ values (for the training set) that would mark the difference between the two classes.

**FIGURE 7:**
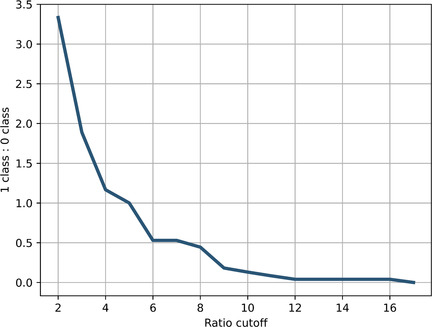
Relationship between the potential cutoff for the ratio between closed and open activities and the class ratio.

Figure 7 illustrates the relationship between the cutoff and the ratio of two label values (classes 0 and 1). At a cutoff value of 5 units, a balance is maintained between the classes of closed‐open form pairs. This prompted us to use this value to construct the model labels. Table S4 (Supporting Information) provides the labels for the Isoform Similarity Model.

To find the optimal Isoform Similarity Model, we performed the same set of experiments as we did for the Cytotoxicity Prediction Model. We tested the use of Morgan, Avalon, Atom, Topological, RDK and Layered fingerprints, MAACS keys, E3FP fingerprints, 3D Mordred descriptors, and MD features. As two main model families, we trained Random Forest classifier from Scikit‐Learn with a max_depth parameter equal to 10 to avoid overfitting and standard MLP with the following architecture: one hidden layer with two times more hidden units than the input feature amount, batch normalization layers and dropout layer with 0.1 dropout probability, ReLU activation function, Adam optimizer with 0.001 learning rate and 0.0005 weight decay and 256 batch size. We also performed feature selection for some experiments. We selected the most valuable features for the model using a variance threshold from Scikit‐Learn with a threshold value of 0.075. For the 2D fingerprints experiment (2D Fingerprints + MLP + Feature Selection), the following features were selected: 126 Morgan, 916 Avalon, 568 Atom, 102 RDK, 134 Topological, 36 Layered fingerprints, and 28 MACCS keys. For the experiment with 3D descriptors (Mordred Features + MD Features + E3FP Fingerprints + MLP + Feature Selection), the following features were selected: 14 MD features (6 for inertia tensor and 1 for PSA for both closed and opened forms), 66 Mordred 3D features, and 1304 E3FP fingerprints. Table 5 shows the results of all experiments for both CV and test sets.

**TABLE 5:** Overview of the experiments for the open‐closed Isoform Similarity Model.

Experiment	Number of features	F1‐score (CV)	F1‐score (test)
Morgan fingerprints + Random Forest	2048	0.398	0.571
E3FP fingerprints + Random Forest	8192	0.533	0.572
Mordred features + MD features + Random Forest	120	0.485	0.607
Morgan fingerprints + MLP	2048	0.498	0.403
2D Fingerprints + MLP	12 622	0.508	0.409
2D Fingerprints + MLP + Feature Selection	1910	0.522	0.433
**E3FP fingerprints + MLP**	**8192**	**0.653**	**0.701**
Mordred features + MD features + MLP	120	0.586	0.693
Mordred features + MD features + E3FP fingerprints + MLP	8312	0.648	0.704
2D Fingerprints + Mordred Features + MD Features + E3FP Fingerprints + MLP	20 934	0.629	0.672
Mordred features + MD features + E3FP fingerprints + MLP + feature selection	1384	0.657	0.694
2D Fingerprints + Mordred Features + MD Features + E3FP Fingerprints + MLP + Feature Selection	3294	0.633	0.685

The following conclusions can be drawn from the results of these experiments:

• 3D descriptors are much more effective than 2D descriptors;
• MLP is a better choice rather than classic ML models;
• The best experiment uses an approach with E3FP fingerprints.

In this approach, since we used a much larger number of features, we decided to expand the hyperparameter search space compared to the previous model. We added tuning for activation functions, increased the range for the number of layers, learning rate, weight decay, and batch size, and introduced optional dropout and batch normalization for each layer instead of a single global parameter as in the previous model. As a result, we had to increase the number of trials for tuning to find the best architecture.

We selected the following hyperparameters for tuning:

• Number of layers: from 1 to 5;
• Activation function: one of [ReLU, LeakyReLU [38], ELU [39], GELU [40]] for each layer;
• For each layer:
• Number of hidden features: one of [8192, 4096, 2048, 1024];
• Optional batch normalization (one of [True, False]);
• Optional dropout (one of [True, False]);
• If the previous parameter is True, the dropout rate is a real number from the range [0.1; 0.5].

Hyperparameters for the optimizer and batch size:

• Learning rate: a real number from the range [0.00001; 0.1];
• Weight decay: a real number from the range [0.00005; 0.005];
• Batch size: one of [32, 64, 128, 256, 512, 1024, 2048, 4096, 8192, 16 384].

The optimizer used was Adam [35], and the tuning process was carried out using the Python Optuna [36] library, applying a Bayesian approach to hyperparameter optimization. F1‐score was chosen as the evaluation metric, and the algorithm aimed to maximize this metric when selecting the best model architecture. The number of trials for the tuning process was set to 500; early stopping was applied, terminating the tuning if no improvement in the evaluation metric was observed for 50 trials. The tuning process executed 357 trials, with the best architecture identified at trial 307.

The best architecture consisted of three layers with 4096, 1024, and 8192 hidden features, LeakyReLU [38] activation function, batch normalization on the second and third layers, dropout on the first layer with a dropout rate of 0.121, a 0.06287 learning rate, a 0.0000584 weight decay, and 8192 batch size. Table 6 provides details of the final model architecture.

**TABLE 6:** The best MLP architecture for the Isoform Similarity Model.

Layer	Output shape	Parameters
BatchNorm1D	[1, 8192]	16 384
Linear	[1, 4096]	33 558 528
LeakyReLU	[1, 4096]	—
Dropout	[1, 4096]	—
Linear	[1, 1024]	4 195 328
BatchNorm1d	[1, 1024]	2 048
LeakyReLU	[1, 1024]	—
Linear	[1, 8192]	8 396 800
BatchNorm1d	[1, 8192]	16 384
LeakyReLU	[1, 8192]	—
Linear	[1, 1]	8 193
Sigmoid	[1, 1]	—

There is no need to estimate the descriptors impact for this model, because the best approach uses only E3FP fingerprints and bits contribution is not informative for future analysis.

The result of this approach is a model capable of predicting whether the open and closed isoforms of the same peptide will have different cytotoxicity or not. Knowing this binary value allows us to conclude the impact of the backbone‐incorporated DAE photoswitch on the peptide structure and dynamics, and consequently, on its biological activity.

The performance of the optimised Isoform Similarity Model, expressed in metrics for all frames (each trajectory frame was considered as an individual input sample for the model) is provided in Table 7.

**TABLE 7:** Metrics for the optimized Isoform Similarity Model (per frame).

Metric	CV	Test
ROC‐AUC	0.653	0.697
Accuracy	0.628	0.690
Recall	0.789	0.765
Precision	0.616	0.666
F1	0.669	0.712

Table 8 provides averaged metrics per frame for each molecule in the test set. In this context, the model predicts either 0 (no difference) or 1 (difference) for each frame in the trajectory (concatenation of the open and closed forms). Subsequently, for each trajectory, the prediction accuracy is calculated and is listed in Table 8 under “Model's accuracy per frame”. Final predicted labels are an average over all frames for a particular molecule passed through the sigmoid to achieve the specific class label. (“Model's accuracy per frame” means the fraction of correctly predicted labels over frames for a particular molecule. For example, if the corrected label for the molecule is 1 (or 0), and for 9 000 out of 10 000 frames, our model predicts 1 (or 0) as well, then the model accuracy per frame for this molecule will be equal to 0.9 or 90%. This can be used to assess how well our model works for a particular molecule.) The last three columns are observed IC_50_ values (µM) in the cytotoxicity experiment against HeLa and their ratio.

**TABLE 8:** Model's accuracy per frame, labelling and experimental cytotoxicities for the test set for the Isoform Similarity Model.

Compound	True label	Predicted label	Model's accuracy per frame	**Closed form IC**_**50**_ **(µM)**	**Open form IC**_**50**_ **(µM)**	**Ratio IC**_**50**_ **(closed/open)**
PS20	0	0	0.961	11	9	1.2
PS4	0	0	0.845	82	41	2.0
PS21	0	1	0.041	100	25	4.0
PS5	1	1	0.598	28	5	5.6
PS9	1	1	0.838	24	3	8.0
PS24	1	1	0.859	300	25	12.0

**FIGURE 8:**
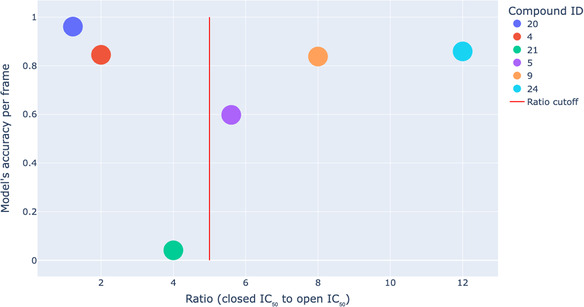
Relationship between the ratio (IC_50_ (closed) to IC_50_ (open)) and accuracy of the Isoform Similarity Model per frame for the test set.

From these results, it can be concluded that the model effectively discerns the absence of differences between the closed and open forms when the ratio between activities is significantly below 5 units (cutoff). This is supported by an accuracy of 0.961 for molecule PS20. However, as the ratio approaches 5 units, the accuracy decreases, and the model makes errors in predicting (as for the pair **PSO21/PSC21**, which has a ratio of 4 units). As the ratio increases, the model becomes increasingly confident in predicting the differences between the closed and open forms, as illustrated in Figure 8. In summary, the model effectively predicts those molecules where differences or similarities between the activities of the closed and open form are substantial, especially when the IC_50_ ratio is significantly above or well below 5 units. Nevertheless, if the ratio is close to 5 units, the model may exhibit errors or a lack of confidence in its predictions, as demonstrated by the pair **PSO5/PSC5**, which has an accuracy of 0.598.

Regarding potential model improvements, expanding the train‐ing dataset could potentially enhance the performance of the model, which might be applicable to other peptides. Incorporating additional peptides into the training data would likely yield a model for predicting the activity difference between closed and open diarylethene photoforms with greater accuracy, tailored to different peptide sets.

Yet another issue should be considered for further improvement of both, Cytotoxicity Prediction Model and Isoform Similarity Model. As we mentioned above, all the open forms of the photoswitchable peptides were arbitrary postulated to have *R*‐configuration at both the asymmetric carbon centers in the DAE fragments. Strictly speaking, it is an oversimplification—in reality, *S*, *S*‐diastereomers could also be formed. As there is no easy way to determine which stereoisomers are formed upon the DAE photoisomerization in each case, choosing the *R*, *R*‐diastereomers for the MD of the open forms in this work was a forced decision. The fact that the models work well despite this oversimplification could be explained: either the *R*, *R*‐diastereomers are indeed formed in reality or the DAE fragment does not contribute significantly to the interaction with the target(s), due to its relatively small size compared to the size of the peptide molecules. The work on this issue is in progress in our laboratory.

### 3.3 Generality of the Isoform Similarity Model

A key question at the outset of this study was whether models based on MD trajectories could be generalized to predict the biological activity or activity differences of photoisomeric pairs of photoswitchable compounds with entirely different mechanisms of action. We questioned whether any model, trained on the activity of a specific set of peptides—macrocyclic diarylethene‐derived membrane‐active gramicidin S analogs—could accurately predict the activity of compounds with different mechanisms of action. By contrast, the Isoform Similarity Model, which utilized concatenated feature vectors from pairs of trajectory frames in the training set, might be effective in predicting activity differences between isoforms. We hypothesized that this prediction could be plausible for the endpoints, even unrelated to the cytotoxicities, on which the model was trained, provided that structural and dynamic similarity represented by the descriptors we choose correlates with biological activity. To test this hypothesis, we used data from our previous studies [41, 42].

**FIGURE 9:**
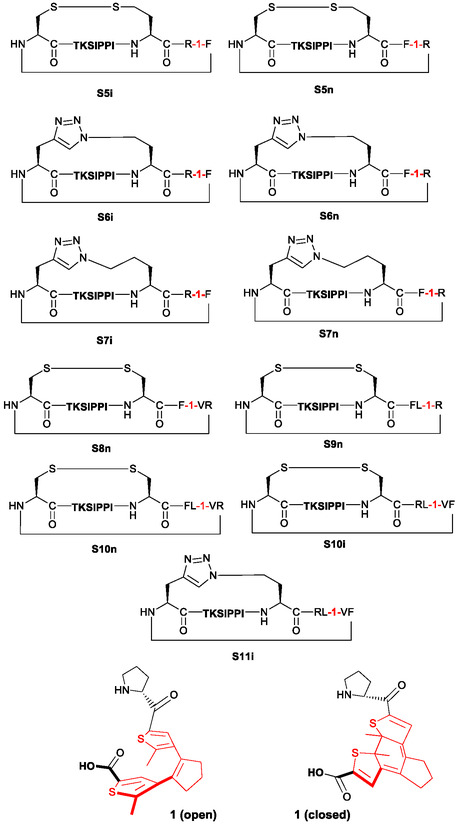
Diarylethene‐based photoswitchable inhibitors of serine protease *Bos taurus* trypsin 1 (T1) described in [41]. The DAE fragment is shown in red.

**FIGURE 10:**
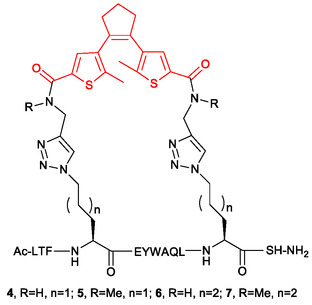
Photoisomerizable modulators of p53/MDM2 interaction described in [42]. The DAE fragment is red‐colored.

The bicyclic peptides described in [41] are photoswitchable inhibitors of serine protease Bos taurus trypsin 1 (T1). They include a diarylethene fragment within one of their rings, as shown in Figure 9. The “stapled” peptides described in [42] (Figure 10)^4^ also contain a diarylethene fragment. They were designed as photoisomerizable modulators of p53/MDM2 interaction. The Ki values for interaction of both the open and closed photoisomeric forms with MDM2 were experimentally determined for these compounds. The data in [42] show that the peptide‐MDM2 interaction is significantly governed by entropic term, so the molecular motion of the target and ligands before and after the interaction play significant role in the interaction. This justifies the use of the Isoform Similarity Model to assess the open‐closed peptide similarity.

Importantly, the biological activity mechanisms of T1 inhibitors and the p53/MDM2 modulators differ from each other and are entirely different from that of the gramicidin S analogs used to train our NN models.

The results of the Isoform Similarity Model evaluation to predict the differences in Ki values between the photoisomeric forms of the peptides described in [41, 42] are summarised in Tables 9 and 10. Although performance in this case is worse than for the test set (Table 8), the Isoform Similarity Model reliably predicted significant (more than fivefold) differences in Ki values for all tested compounds. The model made errors only in five cases, where the activity ratio was below 5 (compounds **S5i**, **S5n**, **S11i**, **5**, and **7**).

**TABLE 9:** Performance of the Isoform Similarity Model for photoswitchable T1 inhibitors.

Compound	**Ratio of K**_**i**_ **(closed/open)**	** True** **label**	Predicted label
**S5i**	1.8	0	1
**S5n**	2.4	0	1
**S6i**	17.5	1	1
**S6n**	8.3	1	1
**S7i**	27.5	1	1
**S7n**	34.7	1	1
**S8n**	24	1	1
**S9n**	6.6	1	1
**S10n**	10.3	1	1
**S10i**	8.8	1	1
**S11i**	3.1	0	1

**TABLE 10:** Performance of the Isoform Similarity Model for photoswitchable p53 inhibitors.

Compound	** Ratio of K**_**i**_ **(closed/open)**	True label	Predicted label
**4**	8.3	1	1
**5**	2.5	0	1
**6**	5.5	1	1
**7**	3.8	0	1

We attribute the strong performance of the Isoform Similarity Model to accounting of both structural and dynamic similarities by the NN. The dynamic similarity may not be immediately apparent to humans, who typically rely on a purely structural and “visual” perspective. Our findings suggest that the NN model effectively captures and computes these “dynamically aware” similarity patterns, which are not easily explained in terms of traditional chemical intuition. Consequently, the calculated similarity level enables the model to predict that for a pair of molecules assessed as similar, their biological activity profiles will also align, regardless of the specific type of activity.

To evaluate whether our multi‐conformer (dynamic, 4D) approach outperforms a comparable method that relies solely on structural (static, 3D) descriptors, we conducted a straightforward experiment. We developed the Isoform Similarity Model using only 3D descriptors calculated from the peptide conformations taken from the first frame of the MD simulation after minimization and equilibration, using E3FP descriptors. The results revealed a test F1 score of 0.67 and an accuracy of 0.5. In comparison, the performance of the 4D model, with an F1 score of 0.712 and an accuracy of 0.69, was noticeably superior, indicating that incorporating dynamic elements significantly enhances the model's predictive capability.

Another potential reason for the strong performance of the Isoform Similarity Model could be a systematic error within the data, leading to an artificial enhancement in model results. However, our investigation did not identify any plausible source of such an error.

It is also possible that the endpoints predicted by the Isoform Similarity Model are interrelated, or that the reported activities of the compounds lack specificity. This hypothesis, however, seems unlikely given existing literature, which suggests that the cytotoxic activity, T1 inhibition, and p53/MDM2‐modulating activity are entirely distinct. Cytotoxic activity primarily involves the disruption of cell membranes in an unspecific manner, whereas T1 inhibition and p53/MDM2 modulation are highly selective and specific.

## 4 Conclusions

The Cytotoxicity Prediction NN Model developed in this study has been optimized to reliably predict IC_50_ values for complex compounds, macrocyclic peptidomimetics. This model was trained using aqueous MD simulation data derived from diarylethene‐based photoswitchable peptides and their cytotoxic activity against a single cell line. Despite the relatively small size of the training dataset, the structural and dynamic information encoded within the MD traces proved sufficient for accurately predicting the cytotoxic activity of related peptides, such as mutants and photoisomers.

In addition, we developed the Isoform Similarity Model, which utilizes MD simulation data from photoisomeric pairs within a membranolytic peptide training set. This model exhibits remarkable efficiency in predicting the activity ratios of photoisomers, particularly distinguishing ratios exceeding 5, even when the biological activity mechanisms of the photoisomers differ significantly from those in the training set.

The Isoform Similarity Model presents significant potential for the rational design of new biologically active photoswitchable peptides or peptidomimetics. For example, it can be used to guide the development of highly efficient photoisomerizable peptides tailored for applications in photopharmacology, thus advancing the design of such compounds.

## Author Contributions

**Serhii Starosyla, Igor V. Komarov** and **Semen Yesylevskyy** formulated the original concept; **Sergii Afonin, Oleg Babii, Taras Voitsitskyi, Roman Stratiichuk, Ihor Koleiev, Volodymyr Vozniak, Nazar Shevchuk, Zakhar Ostrovsky, Alan Nafiiev, Anne S. Ulrich, Aigars Jirgensons** refined the hypotheses and theoretical models used in the work; **Anton Cherednichenko** performed MD simulations, **Taras Voitsitskyi, Roman Stratiichuk, Ihor Koleiev, Volodymyr Vozniak, Zakhar Ostrovsky, Alan Nafiiev** applied the models and collected the final data; all the authors analysed the results and formulated conclusions; **Anne S. Ulrich, Aigars Jirgensons, Igor V. Komarov** managed the project and supervised the work; **Igor V. Komarov** wrote the original draft; all the authors discussed and finalised the text and figures.

## Conflicts of Interest

The authors declares no conflicts of interest.

## Supporting information

Supplementary Material

## Data Availability

The authors have nothing to report.

## Endnotes

1
Compound IDs correspond to the compound numbering in [29].

2
*R*,*R*‐configuration of the DAE fragment in the closed forms of the peptides was set for the MD calculations. This choice was made arbitrary; it may happen that *S*,*S*‐forms are actually formed, and they may have different activity. See the discussion of this problem in Section 3.2.

3
The authors acknowledge the referee who noted the parallels between NB analysis and our approach during the refereeing process.

4
Compound IDs in Figures 9 and 10 correspond to the compound numbering in [41, 42].
