# Supplementary material for: Neural Network Models for Prediction of Biological Activity using Molecular Dynamics Data: A Case of Photoswitchable Peptides
Source: Mol Inform. 2025 Jul 14;44(7):e70001. doi: 10.1002/minf.70001 (PMC12257427; doi:10.1002/minf.70001)
Supplement: Supplementary file 1 — Supplementary Material [file MINF-44-e70001-s001.pdf]

Supplementary Information

Neural Network Models for Prediction of Biological Activity Using Molecular Dynamics Data: A Case of Photoswitchable Peptides

*Anton Cherednychenko, Sergii Afonin, Oleg Babii, Taras Voitsitskyi, Roman Stratiichuk, Ihor Koleiev, Volodymyr Vozniak, Zakhar Ostrovsky, Semen Yesylevskyy, Alan Nafiiev, Serhii Starosyla, Anne S. Ulrich, Aigars Jirgensons, Igor V. Komarov**

Complete set of the compounds used in training and validation of the NN models described in the paper are given in Fig. S1.

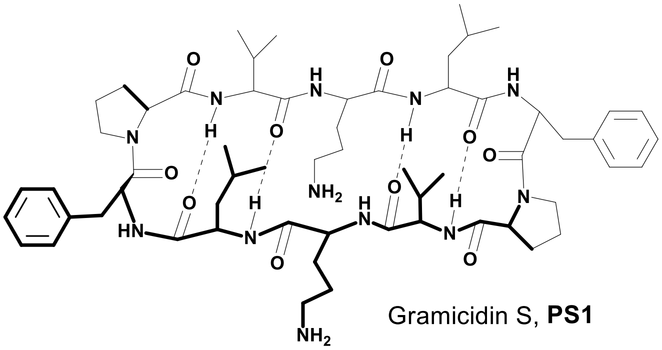

Gramicidin S, **PS1**

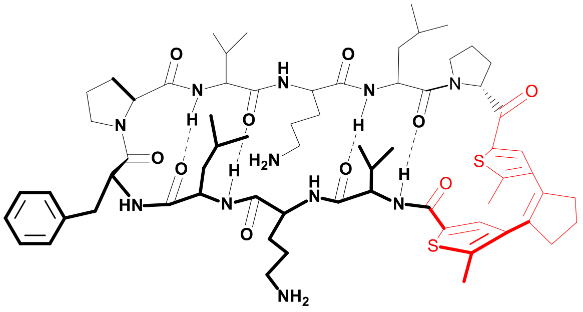

PSO2

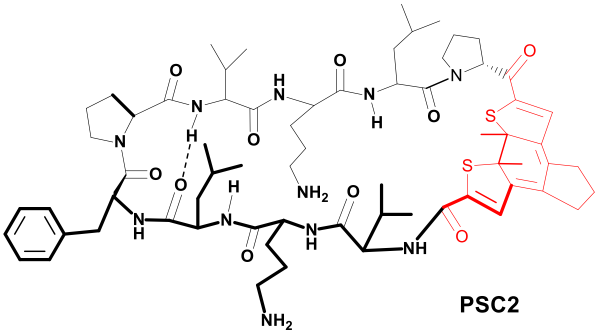

PSC2

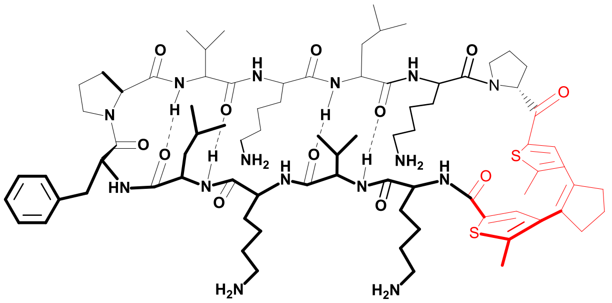

PSO3

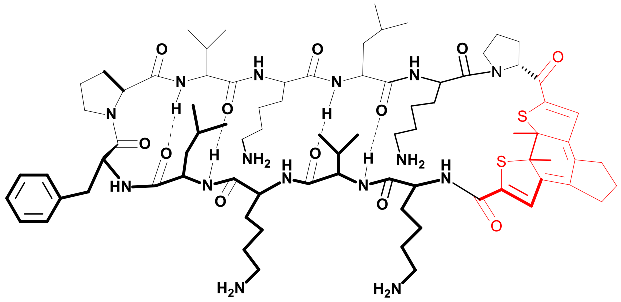

PSC3

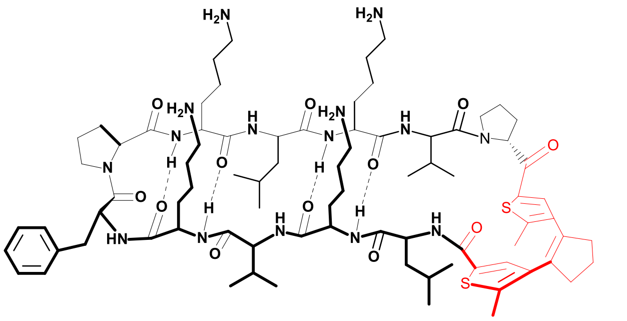

PSO4

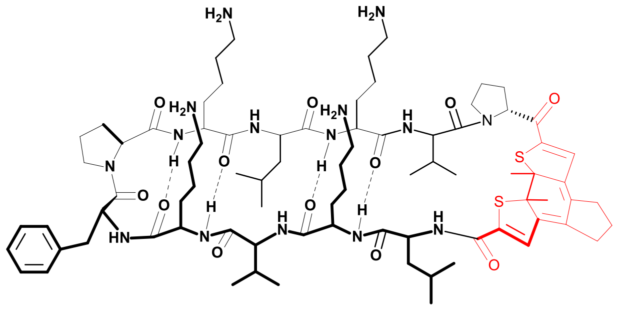

PSC4

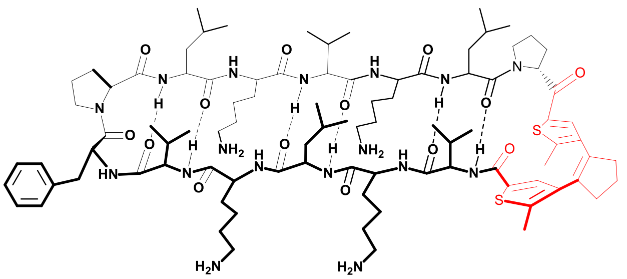

PSO5

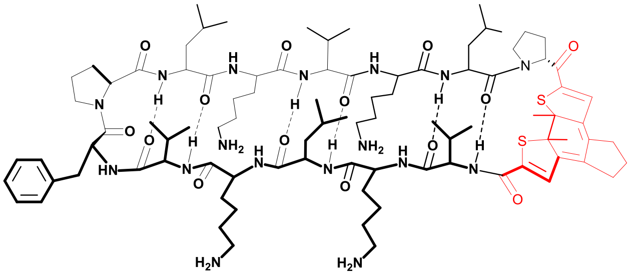

PSC5

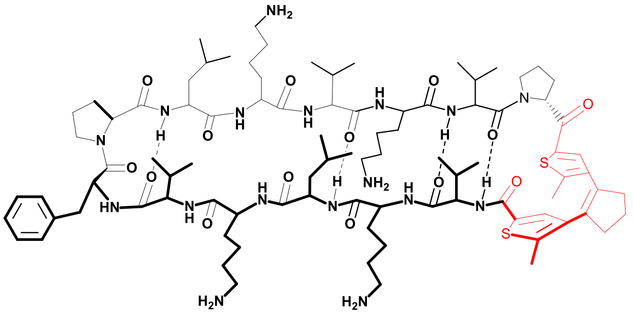

PSO6

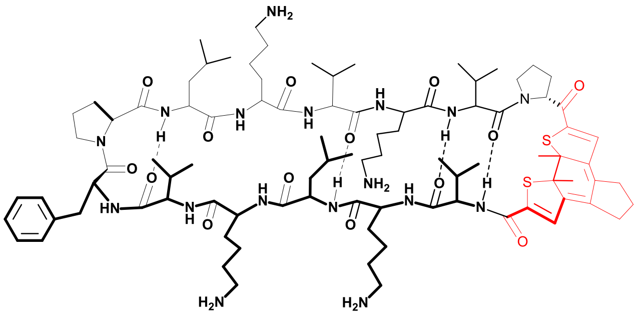

PSC6

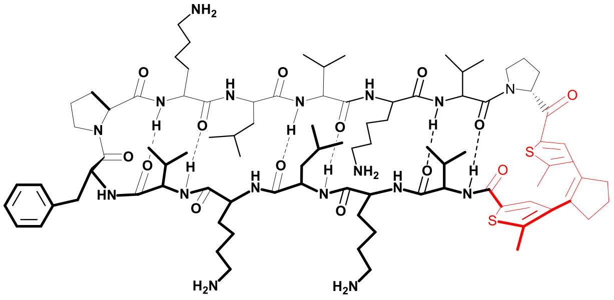

PSO7

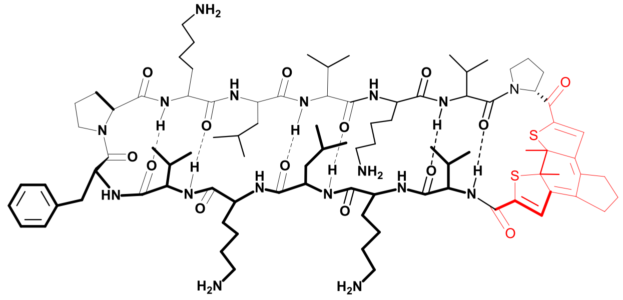

PSC7

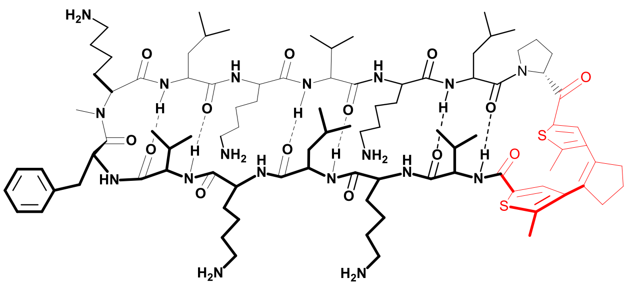

PSO8

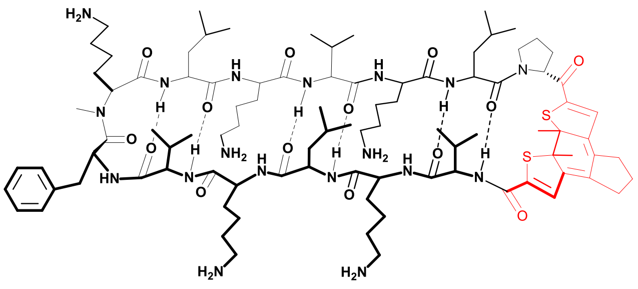

PSC8

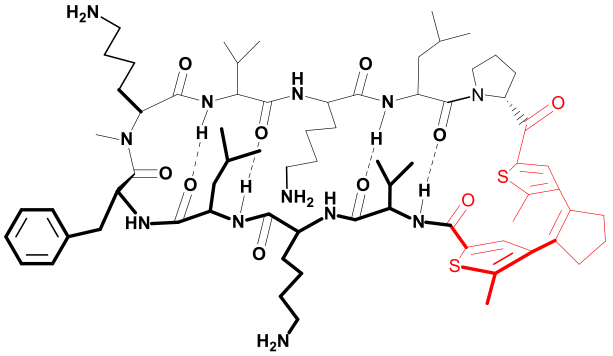

PSO9

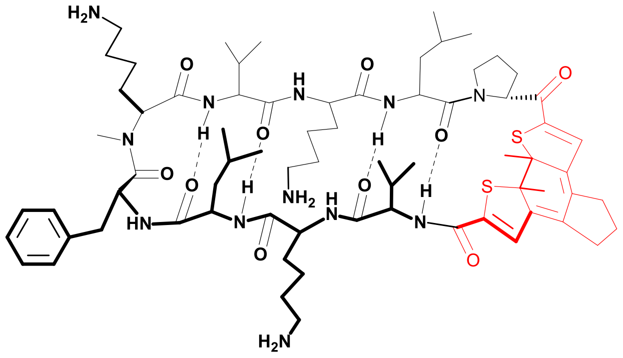

PSC9

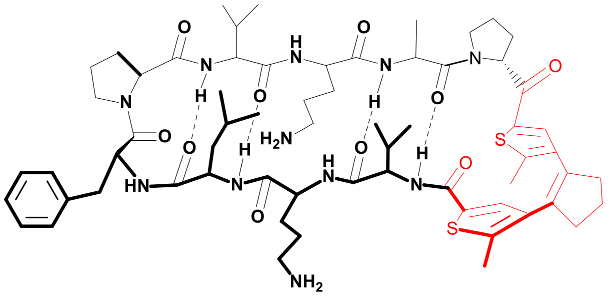

PSO10

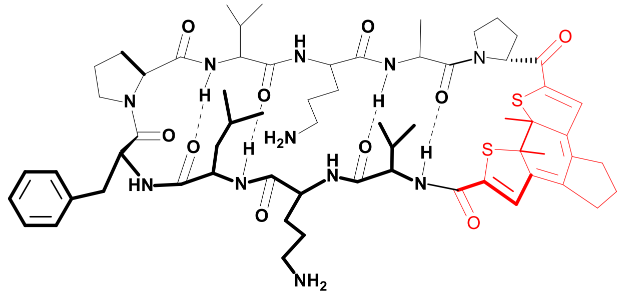

PSC10

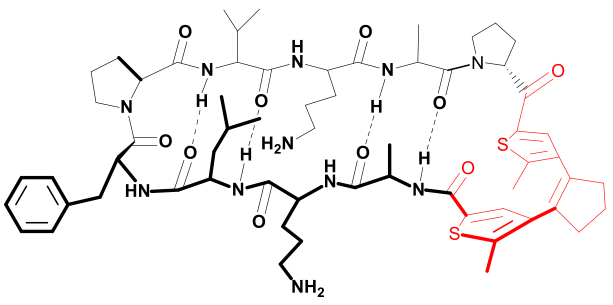

PSO11

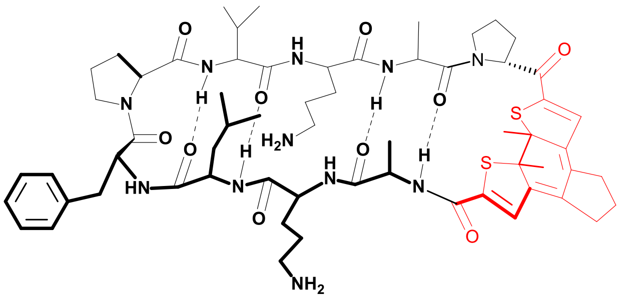

PSC11

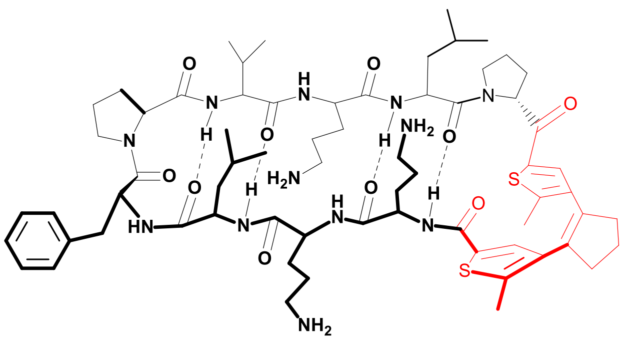

PSO12

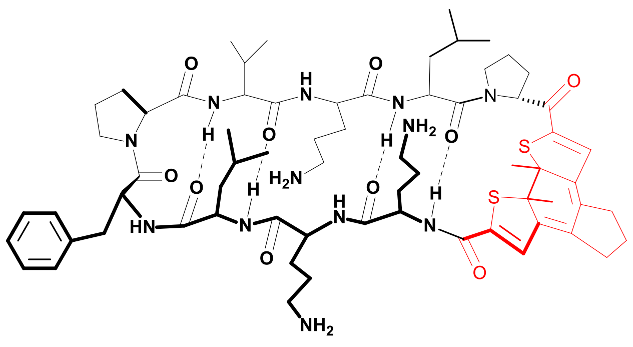

PSC12

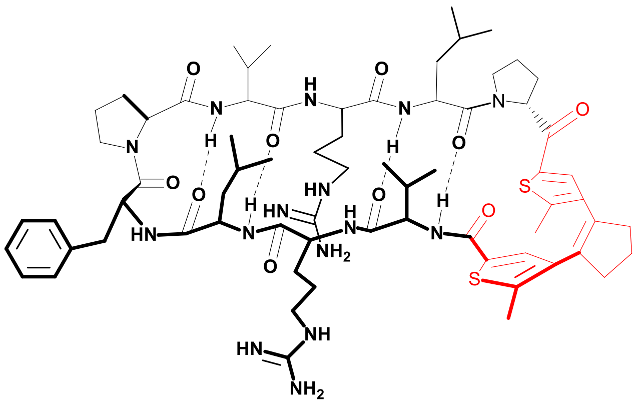

PSO13

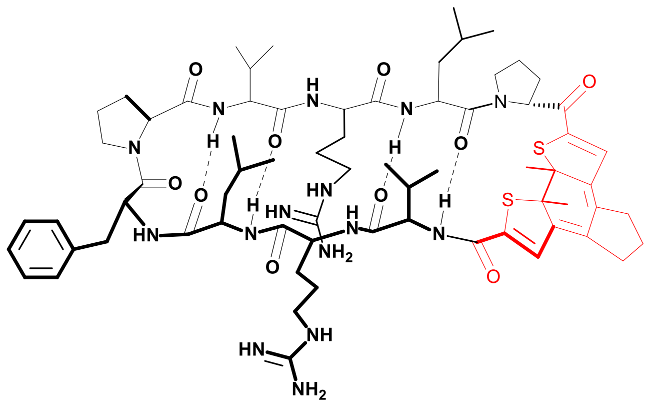

PSC13

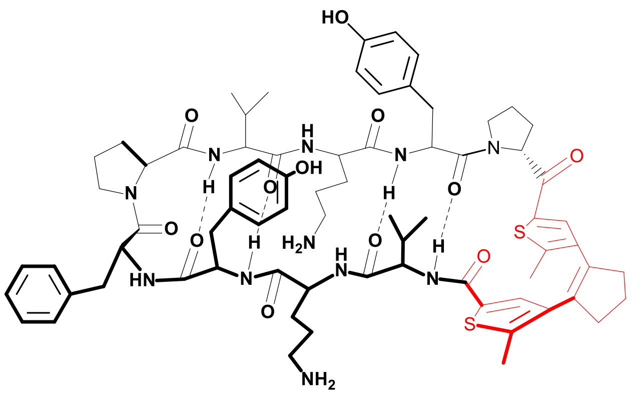

PSO14

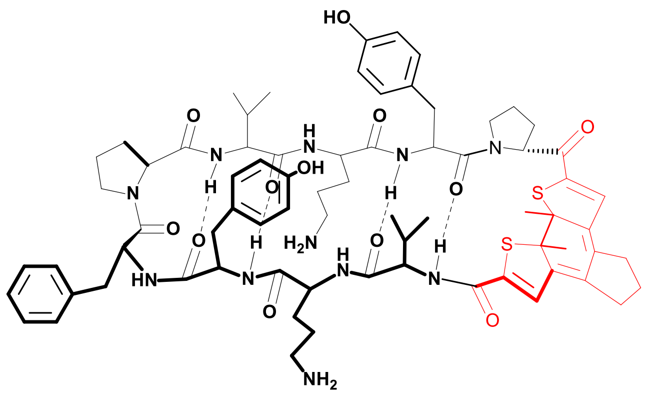

PSC14

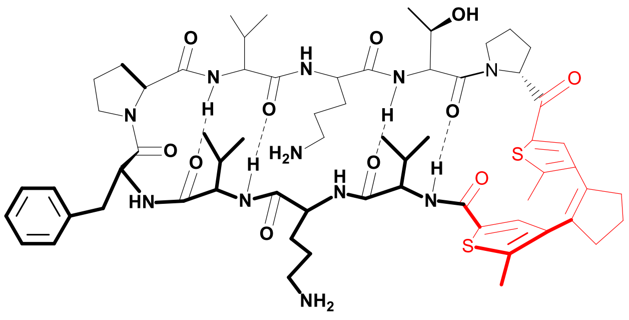

PSO15

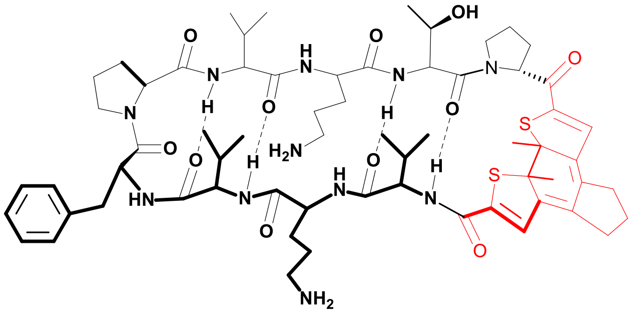

PSC15

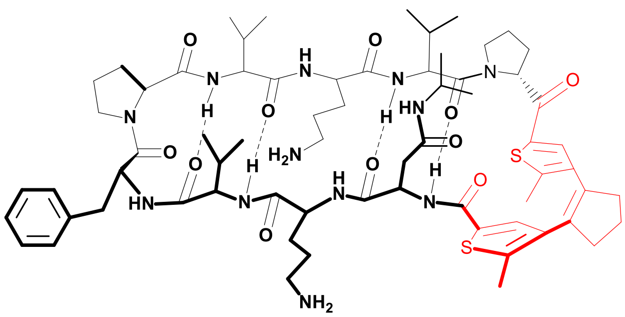

PSO16

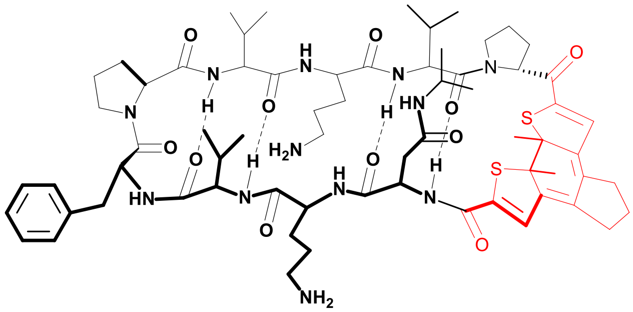

PSC16

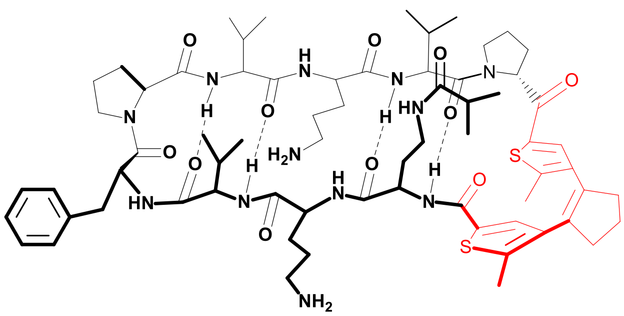

PSO17

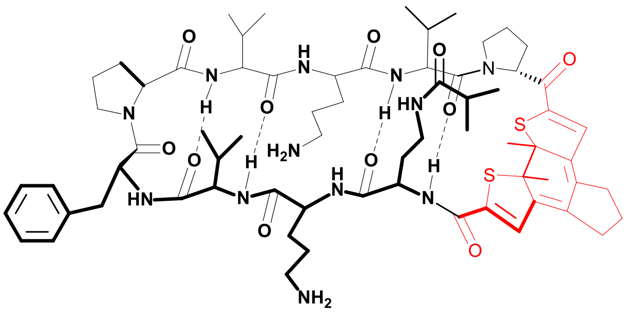

PSC17

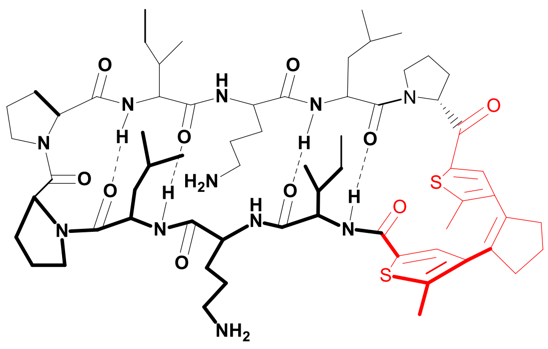

PSO18

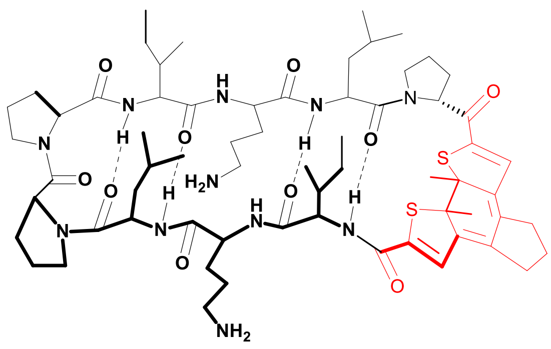

PSC18

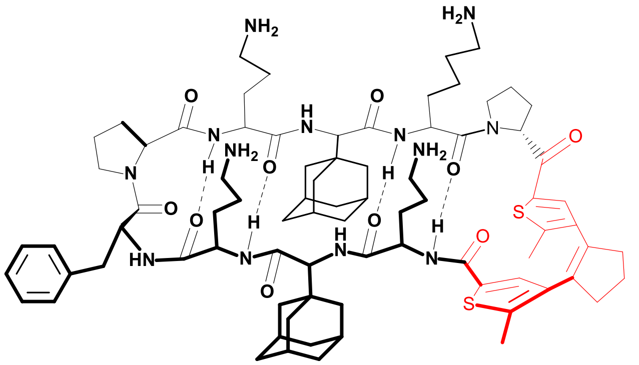

PSO19

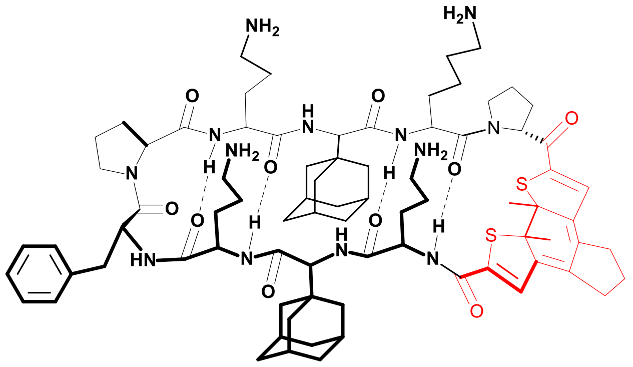

PSC19

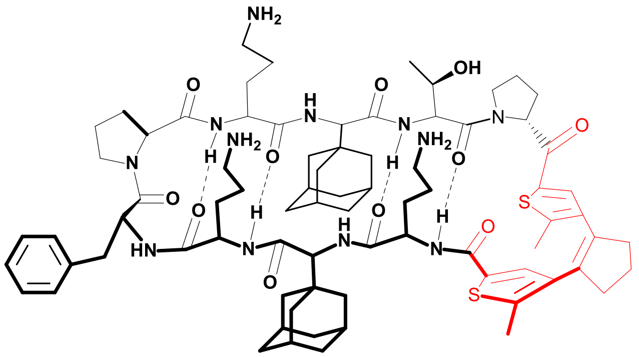

PSO20

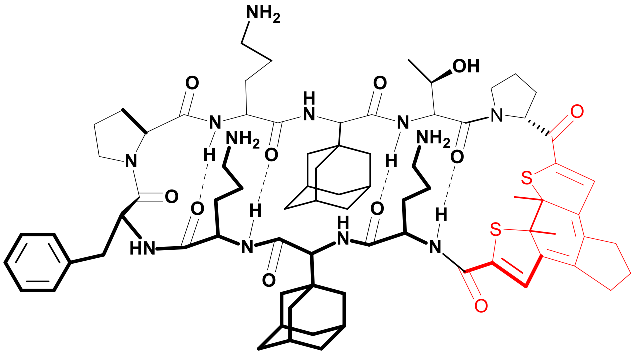

PSC20

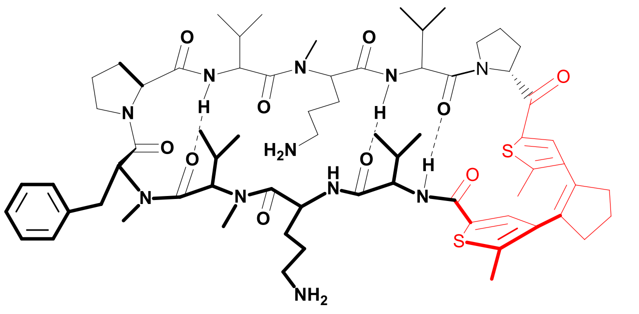

PSO21

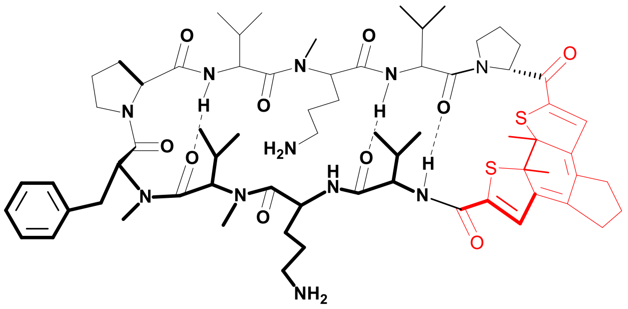

PSC21

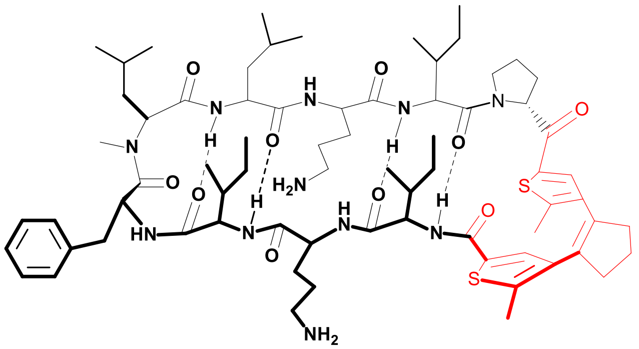

PSO22

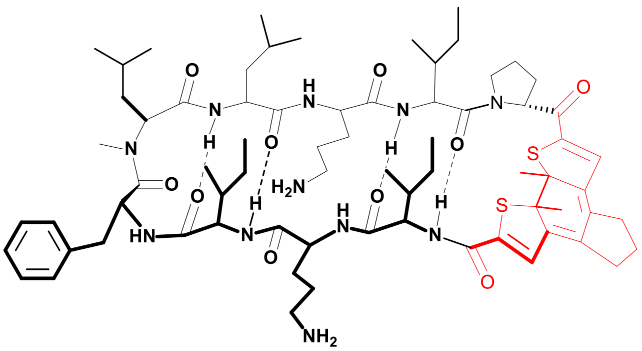

PSC22

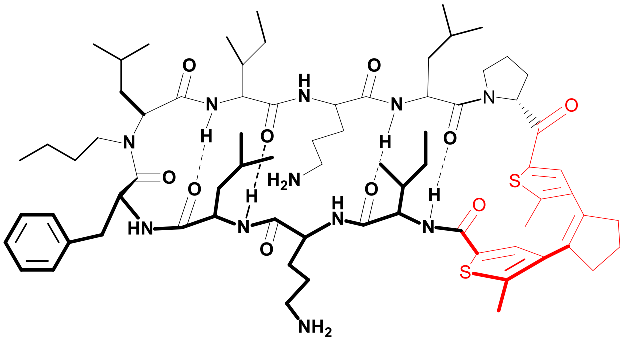

PSO23

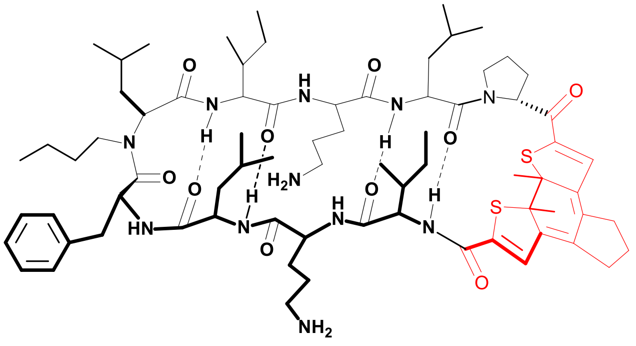

PSC23

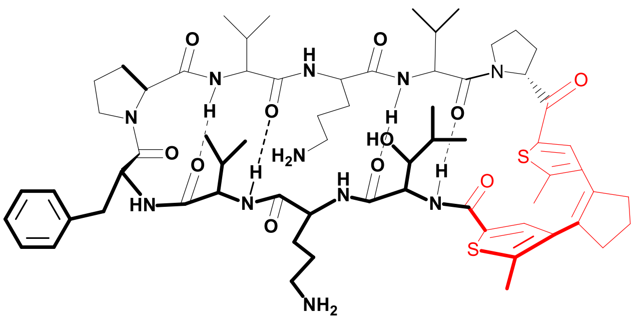

PSO24

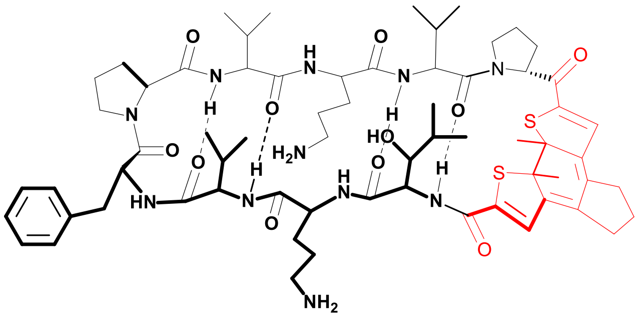

PSC24

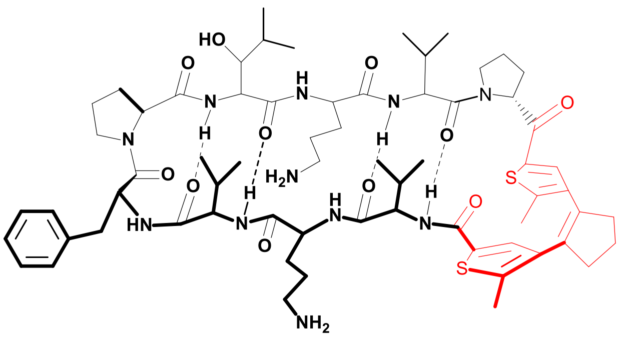

PSO25

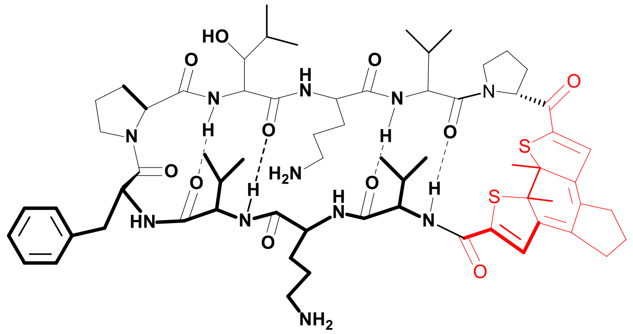

PSC25

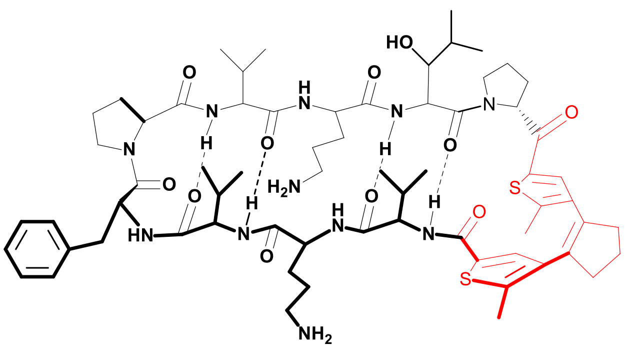

PSO26

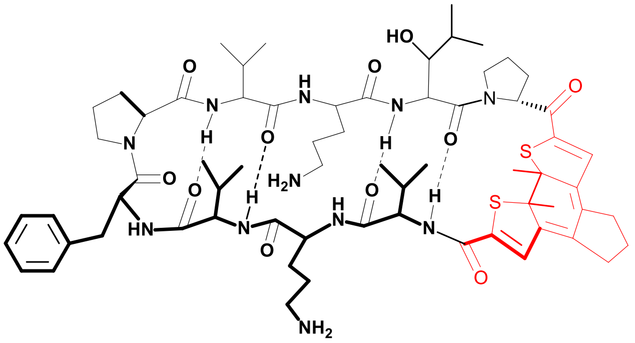

PSC26

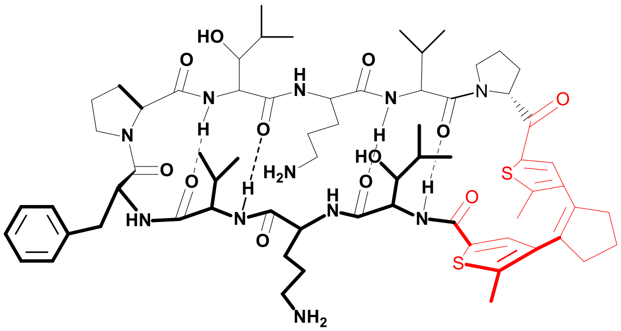

PSO27

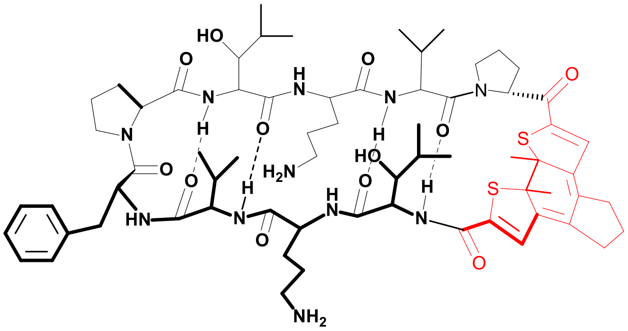

PSC27

Figure S1. Structural formulas of the compounds used for training and validation of the Peptide Activity Prediction Model and Peptide Similarity Prediction Model.

Molecular Dynamics (MD) calculations of peptides.

Twenty-seven closed and open photoswitchable analogs of Gramicidin S were constructed manually. R,R-configuration of the stereocenters of the diarylethene fragment was set in all the derivatives, to ensure uniform comparison. The structures were prepared with protonation states corresponding to physiological pH (7.4) and minimized in the OPLS3e force field, known for its accuracy in modeling peptides, using the PRCG (Polak-Ribier Conjugate Gradient) minimization method [S1].

The system preparation included solvation in explicit TIP3P water, chosen for its efficiency and reliability, with Na+ and Cl− ions added to neutralize the system and mimic physiological ionic strength (0.15M). Default Desmond relaxation protocols [S2], which involve gradual energy minimization and equilibration, were applied prior to MD runs to ensure a stable starting configuration.

Molecular dynamics simulations of peptides were performed using Desmond Molecular Dynamics software from Schrödinger [S2], in the NPT ensemble at 300K using a Nosé-Hoover thermostat and Martyna-Tobias-Klein barostat. Each trajectory was run for 50 ns, a timescale sufficient for the relatively small Gramicidin S derivatives (up to 14 amino acids) to reach stable conformations. Representative data and convergence plots for selected trajectories are illustrated in Fig. S2.

This setup was validated by confirming that the simulation conditions reproduced well the known structure of Gramicidin S [S3], supporting the reliability of the approach for its photoswitchable derivatives.

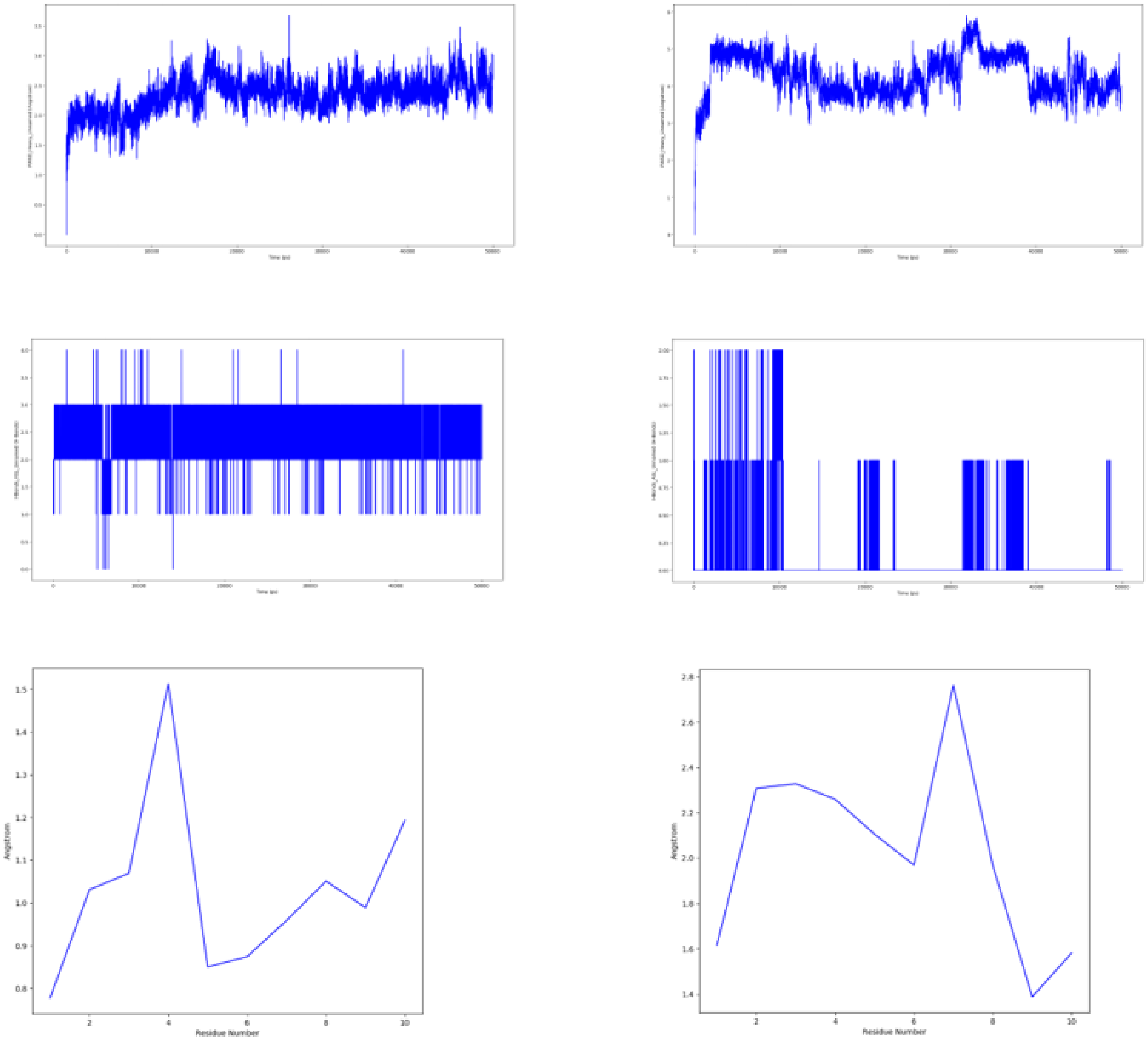

Figure S2. RMSD (upper row), number of H-bonds (middle row) vs the simulation time and residue RMSF (lower row) for ***PSO2*** *(left) and* ***PSC2*** *(right).*

Table S1. Labels for the Activity Prediction Model.

	**Molecule ID**	**IC_{50}**
1	**PSO2**	5
2	**PSC2**	41
3	**PSO3**	41
4	**PSC3**	82
5	**PSO4**	41

6	**PSC4**	82
7	**PSO5**	5
8	**PSC5**	28
9	**PSO6**	9
10	**PSC6**	36
11	**PSO7**	3
12	**PSC7**	18
13	**PSO8**	2
14	**PSC8**	18
15	**PSO9**	3
16	**PSC9**	24
17	**PSO10**	19
18	**PSC10**	39
19	**PSO11**	40
20	**PSC11**	106
21	**PSO12**	6
22	**PSC12**	12
23	**PSO13**	4
24	**PSC13**	35
25	**PSO14**	23
26	**PSC14**	137
27	**PSO15**	77
28	**PSC15**	153
29	**PSO16**	66
30	**PSC16**	196
31	**PSO17**	36
32	**PSC17**	194
33	**PSO18**	6
34	**PSC18**	64
35	**PSO19**	11

36	**PSC19**	11
37	**PSO20**	9
38	**PSC20**	11
39	**PSO21**	25
40	**PSC21**	100
41	**PSO22**	3
42	**PSC22**	25
43	**PSO23**	4
44	**PSC23**	13
45	**PSO24**	25
46	**PSC24**	300
47	**PSO25**	9
48	**PSC25**	150
49	**PSO26**	31
50	**PSC26**	300
51	**PSO27**	61
52	**PSC27**	293

Table S2. Splits for the Activity Prediction Model.

Split name	Train IDs	Val (Test) IDs
**Fold 1**	PSO4, PSC4, PSO6, PSC6, PSO8, PSC8, PSC9, PSO9, PSO11, PSC11, PSC12, PSO12, PSO13, PSC13, PSO15, PSC15, PSO16, PSC16, PSO17, PSC17, PSO21, PSC21, PSC22, PSO22, PSO23, PSC23, PSC24, PSO24, PSC25, PSO25	PSC2, PSO2, PSC5, PSO5, PSC7, PSO7, PSO20, PSC20, PSO26, PSC26
**Fold 2**	PSC2, PSO2, PSO4, PSC4, PSC5, PSO5, PSO6, PSC6, PSC7, PSO7, PSO8, PSC8, PSO13, PSC13, PSO15, PSC15, PSO16, PSC16, PSO17, PSC17, PSC20, PSO20, PSC22, PSO22, PSO23, PSC23, PSC25, PSO25, PSC26, PSO26	PSC9, PSO9, PSC11, PSO11, PSO12, PSC12, PSO21, PSC21, PSC24, PSO24

**Fold 3**	PSC2, PSO2, PSC5, PSO5, PSO6, PSC6, PSC7, PSO7, PSO8, PSC8, PSC9, PSO9, PSO11, PSC11, PSC12, PSO12, PSO15, PSC15, PSO17, PSC17, PSC20, PSO20, PSO21, PSC21, PSO23, PSC23, PSC24, PSO24, PSC26, PSO26	PSO4, PSC4, PSC13, PSO13, PSO16, PSC16, PSC22, PSO22, PSC25, PSO25
**Fold 4**	PSC2, PSO2, PSO4, PSC4, PSC5, PSO5, PSC7, PSO7, PSC9, PSO9, PSO11, PSC11, PSC12, PSO12, PSO13, PSC13, PSO16, PSC16, PSC20, PSO20, PSO21, PSC21, PSC22, PSO22, PSC24, PSO24, PSC25, PSO25, PSC26, PSO26	PSO6, PSC6, PSC8, PSO8, PSC15, PSO15, PSC17, PSO17, PSC23, PSO23
**Train-Test**	PSC2, PSO2, PSO4, PSC4, PSC5, PSO5, PSO6, PSC6, PSC7, PSO7, PSO8, PSC8, PSC9, PSO9, PSO11, PSC11, PSC12, PSO12, PSO13, PSC13, PSO15, PSC15, PSO16, PSC16, PSO17, PSC17, PSC20, PSO20, PSO21, PSC21, PSC22, PSO22, PSO23, PSC23, PSC24, PSO24, PSC25, PSO25, PSC26, PSO26	PSO3, PSC3, PSO10, PSC10, PSC14, PSO14, PSO18, PSC18, PSO19, PSC19, PSO27, PSC27

Table S3. Split information for the Activity Prediction Model.

Data	Samples count	Percentage of data (%)
**All**	520000	100.0
**Train**	400000	76.9
**Test**	120000	23.1
**Train (fold 1)**	300000	57.7
**Val (fold 1)**	100000	19.2
**Train (fold 2)**	300000	57.7
**Val (fold 2)**	100000	19.2
**Train (fold 3)**	300000	57.7
**Val (fold 3)**	100000	19.2
**Train (fold 4)**	300000	57.7
**Val (fold 4)**	100000	19.2

Table S4. Labels for the Peptide Similarity Prediction Model.

Molecule ID	Closed (IC_{50})	Open (IC_{50})	Ratio	Label
**2**	41	5	8.200	1
**3**	82	41	2.000	0
**4**	82	41	2.000	0
**5**	28	5	5.600	1
**6**	36	9	4.000	0
**7**	18	3	6.000	1
**8**	18	2	9.000	1
**9**	24	3	8.000	1
**10**	39	19	2.053	0
**11**	106	40	2.650	0
**12**	12	6	2.000	0
**13**	35	4	8.750	1
**14**	137	23	5.957	1
**15**	153	77	1.987	0
**16**	196	66	2.970	0
**17**	194	36	5.389	1
**18**	64	6	10.667	1
**19**	11	11	1.000	0
**20**	11	9	1.222	0
**21**	100	25	4.000	0
**22**	25	3	8.333	1
**23**	13	4	3.250	0
**24**	300	25	12.000	1
**25**	150	9	16.667	1
**26**	300	31	9.677	1
**27**	293	61	4.803	0

Table S5. Splits for the Peptide Similarity Prediction Model.

Split name	Train IDs	Validation (Test) IDs
**Fold 1**	2, 6, 7, 10, 11, 12, 13, 15, 16, 17, 22, 23, 25, 26, 27	3, 8, 14, 18, 19
**Fold 2**	2, 3, 6, 8, 10, 11, 12, 13, 14, 16, 17, 18, 19, 22, 26	7, 15, 23, 25, 27
**Fold 3**	3, 6, 7, 8, 10, 11, 13, 14, 15, 18, 19, 22, 23, 25, 27	2, 12, 16, 17, 26
**Fold 4**	2, 3, 7, 8, 12, 14, 15, 16, 17, 18, 19, 23, 25, 26, 27	6, 10, 11, 13, 22
**Train-Test**	2, 3, 6, 7, 8, 10, 11, 12, 13, 14, 15, 16, 17, 18, 19, 22, 23, 25, 26, 27	4, 5, 9, 20, 21, 24

Table S6. Split information for the Peptide Similarity Prediction Model.

Data	Samples count	Percentage of data (%)
**All**	26000	100.0
**Train**	20000	76.9
**Test**	6000	23.1
**Train (fold 1)**	15000	57.7
**Val (fold 1)**	5000	19.2
**Train (fold 2)**	15000	57.7
**Val (fold 2)**	5000	19.2
**Train (fold 3)**	15000	57.7
**Val (fold 3)**	5000	19.2
**Train (fold 4)**	15000	57.7
**Val (fold 4)**	5000	19.2

The whole list of Mordred descriptors used in the work:
WPSA1, DPSA1, GRAVp, PNSA1, GeomPetitjeanIndex, PNSA2, WPSA4, DPSA4, PNSA4, PPSA2, WNSA1, PPSA4, WNSA3, WPSA2, DPSA2, MOMI-Y, MOMI-X, MOMI-Z, FNSA3, PPSA5, FPSA1, RPSA, GeomRadius, WPSA5, FPSA3, DPSA5, WNSA2, PNSA5, RNCS, RASA, FNSA1, WNSA4, FNSA2, FNSA4, WNSA5, TPSA, RPCS, FPSA2, TASA, FPSA4, GRAV, WPSA3, DPSA3, PBF, FPSA5, PNSA3, GeomDiameter, PPSA1, GeomShapeIndex, PPSA3, FNSA5)

References

[S1] Perry, Avinoam. “A Modified Conjugate Gradient Algorithm.” Operations Research, vol. 26, no. 6, 1978, pp. 1073–78. JSTOR, http://www.jstor.org/stable/170266. Accessed 9 Mar. 2024.

[S2] Schrödinger Release 2024-1: Desmond Molecular Dynamics System, D. E. Shaw Research, New York, NY, 2024. Maestro-Desmond Interoperability Tools, Schrödinger, New York, NY, 2024.
[S3] Asano, Akiko, and Doi, Mitsunobu. "Crystal Structure of Gramicidin S Hydrochloride at 1.1 Å Resolution." X-ray Structure Analysis Online 35 (2019): 1-2.
